# Use of Social Network Sites for Communication Among Health Professionals: Systematic Review

**DOI:** 10.2196/jmir.8382

**Published:** 2018-03-28

**Authors:** Windy SY Chan, Angela YM Leung

**Affiliations:** ^1^ School of Health Sciences Caritas Institute of Higher Education New Territories China (Hong Kong); ^2^ Faculty of Health and Social Sciences The Hong Kong Polytechnic University Hung Hom, Kowloon China (Hong Kong); ^3^ Centre for Gerontological Nursing School of Nursing The Hong Kong Polytechnic University Hung Hom, Kowloon China (Hong Kong)

**Keywords:** social networking, social media, health communication, Facebook, WhatsApp, professional network, health professionals

## Abstract

**Background:**

Although much research has been done investigating the roles of social network sites (SNSs) in linking patients and health professionals, there is a lack of information about their uses, benefits, and limitations in connecting health professions only for professional communication.

**Objective:**

This review aimed to examine the utilization of SNSs for communication among health professionals in (1) frontline clinical practice, (2) professional networks, and (3) education and training to identify areas for future health communication research.

**Methods:**

This review followed the Preferred Reporting Items for Systematic Reviews and Meta-analyses guidelines. A systematic search of the literature published in the last 10 years (January 1, 2007, to March 1, 2017) was performed in March 2017, using the following electronic databases: MEDLINE via OvidSP, EMBASE, CINAHL Complete, and InfoSci-Journals. The searches were conducted using the following defined search terms: “social media” OR “social network” OR “social network site” OR “Facebook” OR “Twitter” OR “Linkedin” OR “Instagram” OR “Weibo” OR “Whatsapp” OR “Telegram” OR “WeChat” AND “health” OR “health profession.”

**Results:**

Of the 6977 papers retrieved, a total of 33 studies were included in this review. They were exploratory in nature, and the majority used surveys (n=25) and interviews (n=6). All retrieved studies stated that SNSs enhanced effective communication and information sharing. SNSs were used for supporting delivering of clinical services, making referrals, and sharing information. They were beneficial to network building and professional collaboration. SNSs were novel tools to enhance educational interactions among peers, students, instructors, and preceptors. The application of SNSs came with restraints in technical knowledge, concerns on data protection, privacy and liability, issues in professionalism, and data protection.

**Conclusions:**

SNSs provide platforms facilitating efficient communication, interactions, and connections among health professionals in frontline clinical practice, professional networks, education, and training with limitations identified as technical knowledge, professionalism, and risks of data protection. The evolving use of SNSs necessitates robust research to explore the full potential and the relative effectiveness of SNSs in professional communication.

## Introduction

### Background

Social network sites (SNSs) are Web-based services that allow individuals to construct a proﬁle and build a network of connections with other users within the system [1]. Since their introduction, SNSs have become integrated into the daily practices of millions of users. With the evolving technologies in mobile-based platforms and apps, SNSs are currently constructed as Web 2.0 Internet-based apps [2].

The world's current largest social network, Facebook, has engaged more than 2.01 billion users worldwide [3,4]. Twitter, with more than 330 million of monthly active users, has become essential to scientific conferences, gaining them publicity via sharing real-time proceedings or live-tweeting [5]. SNSs provide platforms for users to share their own content, react, or add comments on the content posted by other users. They help strangers to be connected based on their common interests, activities, identities, or professions. LinkedIn, with more than 530 million members in over 200 countries and territories, focuses on business connections and industry contacts for employers and working professionals. It allows users to enhance their connectedness in their areas of expertise [6]. SNSs differ from traditional broadcast media in supporting networking by information and communication technologies. WhatsApp Messenger brings free, cross-platform communication beyond text-only messages to more than 1 billion people in over 180 countries [7].

Availability and preferences of SNSs vary across countries. Facebook is the top worldwide yet, in some countries, such as Indonesia, Instagram has taken its place, and some African territories prefer LinkedIn [8]. In China, where some SNSs are not available, QZone is the top social network. VKontakte and Odnoklassniki, which are both controlled by Russia’s Mail.Ru group, have also gained ground in Russian territories [8].

SNSs are widely used in health communication and research [9] and provide platforms to the public to access health information and to seek support if needed. A new dimension to health care was created to enable the public, patients, and health professionals to communicate about health issues and to give them the possibility of improving health outcomes [10]. In a meta-analysis, SNS interventions were found to be effective in changing health behavior–related outcomes in which the predominant health domain was fitness related (eg, weight loss and physical activity) [11]. Emerging evidence support using SNSs among health professionals to develop virtual communities for sharing domain knowledge [12].

### Objective

Most current literature reviews have focused on the roles of SNSs in linking patients and health professionals [9,10,13].Nevertheless, there is a lack of information about the uses, benefits, and limitations of SNSs in connecting health professions only (excluding the involvement of patients). This systematic review aims to examine the utilization of SNSs for communication among health professionals in (1) frontline clinical practice, (2) professional networks, and (3) education and training to identify important areas for health communication research in the future. In the context of this review, frontline clinical practice refers to the delivery and operation of health services; professional networks refer to the interactions and relationships of a professional nature rather than personal interactions; and education and training are meant to be the training of students and professional development in the health care field.

## Methods

### Search Strategy

This review followed the Preferred Reporting Items for Systematic Reviews and Meta-analyses (PRISMA) guidelines [14]. A systematic search of the literature published in the last 10 years (January 1, 2007, to March 1, 2017) was performed in March 2017, using the following electronic databases: MEDLINE via OvidSP, EMBASE, CINAHL Complete, and InfoSci Journals.

SNSs itself has not been defined as a medical subject headings (MeSH) to optimize retrieval of relevant papers, “social media” (a MeSH term) is used in the search because SNSs are considered as a subset of social media [9]. As the number of SNSs being used rises continuously, the search terms were limited to the top most frequently used ones [3]. The searches were performed using the following search terms: “social media” (a MeSH term) OR “social network” OR “social network site” OR “Facebook” OR “Twitter” OR “Linkedin” OR “Instagram” OR “Weibo” OR “Whatsapp” OR “Telegram” OR “WeChat” AND “health” (a MeSH term) OR “health profession” (a MeSH term).

Initial screening of the studies, based on the information contained in the titles and abstracts, was undertaken independently by 2 reviewers. If a decision on inclusion or exclusion could not be reached, the full text was retrieved. The full texts of the shortlisted papers were then assessed independently by 2 reviewers. The reference lists of relevant papers were also screened for eligible papers. The reviewers met to discuss studies for inclusion and to reach consensus. If there was a discrepancy, a third reviewer was consulted.

### Study Inclusion and Exclusion Criteria

This review included all study designs to identify the best evidence available to address the research objective. Studies were included in this review if they (1) focused primarily on communication interactions between and among health professionals about health issues using SNSs and (2) studied the uses, benefits, or limitations of SNSs.

Studies were excluded from this review if they (1) were not in English, (2) were reviews, reports, abstracts only, letters, or commentaries, (3) focused primarily on the communication between public or patients and health professionals, or for personal uses, (4) described the use of SNSs primarily with a marketing or advertising focus, (5) studied non-SNS types of social media (eg, websites, short message service, emails, hospital information systems, and electronic health record systems), or (6) were not available as full text in the final search.

### Data Extraction, Synthesis, and Evaluation

A computer-based form was created for data extraction. The data collected included first author, year, country, study type, number of participants, health profession(s) involved, type(s) of SNSs, functions of the SNSs (eg, for education, data sharing, continuous professional development), controls and their characteristics (if applicable), and primary outcome measures (and secondary outcome measures if they were highly relevant). The Critical Appraisal Skills Programme (CASP) appraisal tools were used to evaluate the quality of the reviewed studies. They can be used to critically appraise the evidence of a wide variety of settings and designs (eg, qualitative studies or studies using mixed methods). Each CASP tool consists of 3 sections, and each section is designed to assess different domains of a primary study (the internal validity of the instruments used in the primary study, the results, and the relevance of the findings to practice) [15,16]. The Quality Assessment Tool for Observational Cohort and Cross-Sectional Studies developed by the National Heart, Lung, and Blood Institute was used to evaluate the quality of the quantitative studies [17]. Two reviewers assessed the quality of the included studies independently. If necessary, a third reviewer was involved in settling disagreements.

### Ethics Approval and Consent to Participate

This was a systematic review with no data collected from human subjects. Ethical approval was not needed.

## Results

### Findings

**Figure 1:**
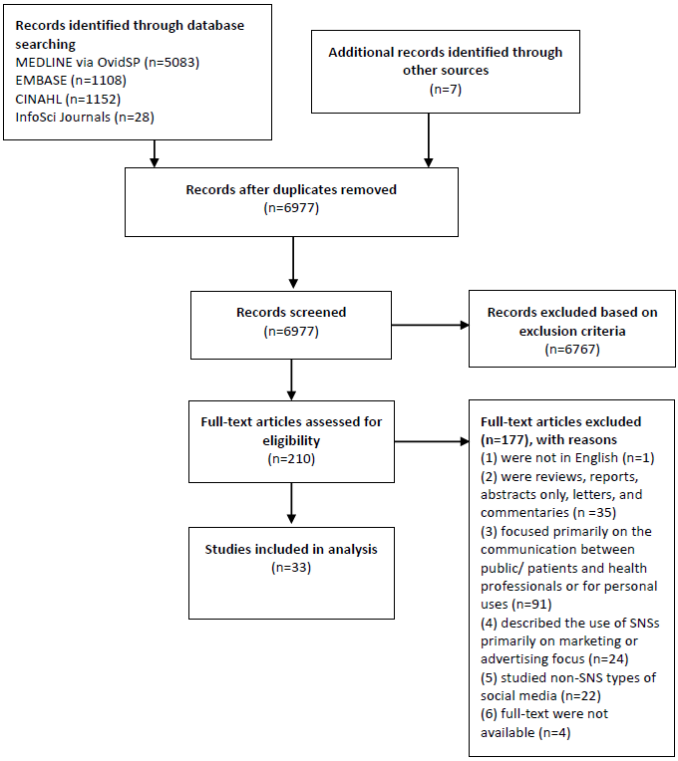
Literature search following Preferred Reporting Items for Systematic Reviews and Meta-analyses (PRISMA) guidelines. SNS: social network site.

Figure 1 shows the searching process and how the studies were included in this review. The literature search retrieved 6977 papers. Their titles and abstracts were screened, and those that did not meet the inclusion criteria were removed. Duplicated titles were also removed. Full texts of 210 papers were assessed for eligibility. A total of 33 studies were finally included in this review. Details of the studies, including study design, study objective, health professionals involved, measurements, SNSs evaluated, and conclusions, are summarized in Multimedia Appendix 1. The studies (n=177) that were excluded are shown in Multimedia Appendix 2, along with the reasons for their exclusion.

### Characteristics of the Reviewed Studies

Among the 33 included studies, more than half of the reviewed studies (n=19) were published in recent 2 to 3 years (between 2015 and 2017). The studies were conducted in 11 countries, the majority being based in the United Kingdom (n=9), the United States (n=12), and Canada (n=4). Other countries with one study included were Australia, China, France, Israel, Saudi Arabia, Malaysia, Singapore, and Turkey (Multimedia Appendix 1). Participants in the reviewed studies were from diverse health professions (Table 1). On many occasions, more than one health profession was involved in the studies evaluating the use of SNSs in clinical practice. Two studies were conducted in large multidisciplinary communities of practice [18,19]. Physicians, including medical and surgical doctors, were involved in about two-thirds of the studies (n=19). Students and trainees were involved in 7 studies in which the uses of SNSs in education and training were evaluated [20-26].

### Assessing the Quality of the Studies

Overall, the quality of studies was satisfactory. Most of the reviewed studies met the criteria in checklists (Multimedia Appendix 3). All studies were exploratory in nature, and the findings were often descriptive. Among the 33 studies, 12 were quantitative [20,21,27-36], 5 qualitative [18,37-40], and 16 used mixed methods [19,22-26,41-50].

No randomized clinical trials (RCTs) were included in this review. No head-to-head comparisons of the relative effectiveness of SNSs could be identified. Researchers often used more than one approach in examining the roles of SNSs and their outcomes. Most studies used surveys (n=25) [19-23,25-36,42-45,47-50]. The questionnaires adopted in the surveys were mostly developed by the researchers. No validated scale was used for surveying the use of SNSs among health professionals. Therefore, conducting a meta-analysis was not possible in this review.

In most of the mixed methods studies, researchers conducted surveys and then analyzed the messages (or communication) in the SNSs. This method is called “content analysis.” Researchers also analyzed the characteristics of SNS users and the context of their communications and SNSs metrics, such as the number of messages, posts, tweets, likes, and followers. Five studies conducted one-on-one interviews [24,37,39,41,46], and one used focus group interviews [44]. Thematic analysis was used in these studies, with key themes being identified from the content of the communications (eg, WhatsApp messages) and user comments.

### Uses and Benefits of Social Network Sites for Professional Communication

The 33 included studies involved a range of SNSs. In 11 studies, the authors conducted cross-sectional surveys or interviews to examine participants’ utilization of any types of social media and SNSs in the broad sense, without concentrating on any particular type of SNS. Among the rest of papers, the most reported SNSs are Twitter, Facebook, WhatsApp Messenger, and LinkedIn. Table 2 describes the types of SNSs studied. All the studies investigating the use of Twitter and Facebook were conducted in North America and the United Kingdom, and those studying WhatsApp Messenger were based in the United Kingdom, the Middle East, and Asia (Multimedia Appendix 1). The one evaluating Sina Weibo was based in China.

Predictors of use of SNSs for professional purposes were often examined by researchers. The positive predictors identified include younger age (20-39 years), fewer years of professional experience (0-10), and lower rank, such as residents and nonconsultants [36,44,48,50]. All retrieved studies stated that SNSs enhanced effective communication and information sharing among health professionals. Participants in the reviewed studies appreciated SNSs as user-friendly, free, and fast tools for communication [24,31,38,45]. The utilization and benefits of SNSs for communication among health professionals in (1) frontline clinical practice, (2) professional networks, and (3) education and training are examined in the following paragraphs.

**Table 1:** Types of health professions included in the reviewed studies.

Health profession	Number of studies^a^
Medical (physicians, specialists, surgeons, and medical students)	20
Pharmacy (pharmacists, pharmacy students, and faculty)	8
Nursing (nurses and student nurses)	4
Multidisciplinary community of practice	2
Forensic occupational therapy	1
Public health	1
Radiology	1

^a^A study could include more than one type of health profession.

**Table 2:** Types of social network sites.

Tool or app	Number of studies^a^
Twitter	8
Facebook	7
WhatsApp	6
LinkedIn	2
Sina Weibo	1
Yahoo online discussion group	1
Web 2.0 (tools not specified)	1
Any types of social media or network site	11

^a^More than 1 social network site was involved in some studies.

#### Uses and Benefits of Frontline Clinical Practice

In the delivery and operation of health services, SNSs are used as channels for communication within clinical teams [27,43,46,49], for seeking clinical consultation or making referrals to consultants or specialists [30,38], for disseminating clinical guidelines, and for promoting awareness of the guidelines among practitioners [32]. WhatsApp Messenger was used when instant responses and actions were required within the framework of the same institute [27,38,46,49]. In a multisite family health team involving many members scattered throughout a territory, Facebook was selected as a tool for communication, collaboration, and informal knowledge exchange [43].

The key benefit was that SNSs being the convenient and efficient channels for information sharing had no restriction by locations or office hours. They were effective in creating a complex, longitudinal stream of information and multimedia files [19]. Photographic and diagnostic images, text messages, videos, and voice messages (eg, rhythm sounds in the monitor worn by patients) were easily shared via WhatsApp messages [27,30,38,46,49]. SNSs allowed the sharing of messages with multiple recipients, which shortened the time for processing. In Wani et al’s study (2013), participating physicians and consultants commented that WhatsApp Messenger was a fast and effective method for the team to evaluate patients and to complete academic endorsement [49]. It was also claimed that it helped to flatten the hierarchy within a clinical team [46]. Johnston et al (2015) and Wani et al (2013) noted that WhatsApp Messenger continued to be the communication system used within the teams after the completion of studies [46,49].

#### Uses and Benefits of Professional Networks

SNSs were used to build and strengthen interactions and relationships of a professional nature. They facilitated connections and collaborations among practitioners of the same health profession [30]. SNS users can strategically search for and join groups of their communities or common interests, such as professions and research areas, that enhanced network building among health professionals of diverse backgrounds but with the same interests, connecting them beyond the scope of their usual practices [18,45].

For instance, the formation of impressive networks among Twitter #hcsmca community members not constrained by professional status was revealed in the social network analysis performed by Gruzd and Haythornthwaite (2013) [18]. In Goff et al’s study (2016), the Twitter group engaged plentiful professionals interested in infectious diseases and antimicrobial stewardship topics [42]. The LinkedIn group, “Hand Surgery International,” demonstrated a remarkable gain in membership, up to 4106 in 4 years. The building of this community of practice took place beyond geographical limitations [45].

The establishment of professional networking and making new contacts was one of the most favorable benefits brought by SNSs [28,37,45]. Professionals can also create a professional online presence, increasing the number of their followers and having a greater impact on readership and content dissemination [29]. Users have a high level of control over the content that they read, listen to, watch, and follow. Among the interviewees in Benetoli et al’s exploratory study (2016), Facebook was preferred over other SNSs for professional purposes because of its popularity, simplicity, and versatility [37].

#### Uses and Benefits of Education and Training

SNSs were used as novel tools for teaching, learning, and enhancing educational interactions among peers, students, instructors, and preceptors [20-23,25,26,47]. Twitter and Facebook were used in course assignments and projects. They were found to be useful, straightforward educational tools to supplement and enhance students’ learning experience [20,21,25]. The utility, feasibility, and acceptability of WhatsApp Messenger in supplementing “problem-based learning” was clearly indicated in the study by Raiman et al (2017) [24]. When used to support teaching and learning, SNSs encouraged interactivity in both peer and academic support [22,23,26]. The applications fostered a positive social atmosphere, generating learning opportunities outside the classroom [24]. They enhanced the construction of students' own learning and the continuation of their engagement in development [20]. Reames et al (2016) concluded that SNSs positively influenced the educational experience and engagement of students [25].

Health professionals can stay abreast of news and information pertaining to their professional interests by following or subscribing to updates in SNSs [21,28,29,33]. For instance, the latest clinical information and real-time surveillance data on an infectious outbreak could be released ahead of peer-reviewed published papers [42]. Facebook and Twitter aided promoting professional development [41,42,50] and also facilitated outreach from a scientific conference, allowing active participation via communication during the conference [19].

### Limitations of Social Network Sites for Professional Communication

Some drawbacks come with the utilization of SNSs. How to operate the SNSs smoothly was a challenge to some health professionals [28,29,36]. In Nikiphorou et al’s study (2016), 30% of non–social media users justified not using SNSs because of lack of knowledge on how to do so [29]. Patel et al (2017) also pointed to unfamiliarity with the technical aspects of SNSs as one of the obstacles to their utilization.

Hesitations on the use of SNSs included concerns regarding data protection, patient privacy, and liability [28,29,43,44]. In a survey, more than half of the respondents were uncertain regarding the procedures or mechanisms for archiving or backing up data [30]. Although WhatsApp Messenger was successfully integrated into the operations of clinical teams, members were concerned that WhatsApp conversations could be regarded as medical records [49]. Fuoco and Leveridge (2015) raised the controversy of whether medical regulatory bodies should monitor the social media activities of health professionals [31]. Nonetheless, whether there was any institutional policy regarding transfer of personal medical information by SNSs was seldom mentioned in studies.

The border between the professional and personal spheres of SNS use was blurred to many health professionals [39]. Exposure of one’s private life was one of the risks of using SNSs that contain detailed personal profile [22,29]. Some health professionals had concerns over the stigma of unprofessionalism and a negative impact on their reputation from the use of SNSs [28,29]. Academic faculty members worried whether being “friended” on Facebook or “followed” on Twitter would blur the boundaries of the instructor-student relationship [33]. On the other side, students said that they felt revision anxiety because their module leaders could read about their personal lives on Facebook [22]. SNSs often provide instant messaging functions. Concerns about the intrusiveness and pushiness of messages, particularly after office hours, were raised by members of clinical teams that used WhatsApp Messengers [24,38].

The implementation of SNSs was not found to be beneficial or effective to participants in all the reviewed studies. Although more than 80% of students agreed that the Sina Weibo improved communication, one-fourth felt that collaborative learning was not effective [26]. Reluctant participation was observed in the use of Twitter designed for enhancing the educational experience of a clerkship. Only 8% of respondents (5 of 62) agreed that Twitter could increase their clerkship engagement [25]. It was proposed that the reluctance was due to the one-way flow of information. In Maisonneuve et al’s study (2015), participants checked (or read) SNS content more often than they posted, and the exchanges on SNSs were limited [39]. Gruzd and Haythornthwaite (2013) concluded that leadership and members' participation were crucial for the effectiveness of online networks [18].

## Discussion

### Principal Findings

The 33 included studies in this review provided evidence that SNSs have been developed as useful platforms for communication among health professionals with significant benefits in the frontline clinical practice, professional networks, and education and training.

Numerous benefits of using SNSs were identified. SNS users in the reviewed studies considered SNSs as user-friendly, easy-to-use, free, and fast tools for communication [24,31,38,45]. In frontline clinical practice, SNSs were efficient in transferring a stream of information and multimedia files instantly to multiple recipients. This highly facilitated the communication among members of the service units or teams [19,27,30,38,46,49]. In building professional networks, SNSs connected professionals beyond the scope and geographical locations of their usual practices [18,42,45]. Users were benefited in making new contacts and expanding their networks [28,37,45]. As tools for education and training, SNSs were useful in generating learning opportunities and enhancing interactions among peers, students, instructors, and preceptors [20-23,25,26,47]. They also promoted update of news and professional development [21,28,29,33,41,42,50].

The merit of SNSs in facilitating interactions, sharing of information, and promoting connections among health professionals is well illustrated in this review. Compared with the findings of the reviews that examined the uses of SNSs between the public and health professionals, this review added value by summarizing the benefits of SNSs in communication among health professionals [9-11,51]. With the increasing use of SNSs, there will be further opportunities to use this efficient tool for professional communication.

In this review, the most reported SNSs were Twitter, Facebook, WhatsApp Messenger, and LinkedIn. Twitter and LinkedIn are robust in expanding a user’s connection because users can easily follow their targets without disclosing much private details or requesting authorization [5,6]. Facebook is designed to share one’s personal profile with “friends”; hence, it may disclose more personal details. It was best used when building and strengthening a community among a group of known people, such as members of the National Physicians Alliance [41], and large cohorts of students [21]. WhatsApp Messenger was appraised as an efficient and easy-to-use app for communication in clinical teams or for linking up students and instructors [24,27,30,38,46,49]. However, its use was constrained within an established framework or a group of recipients because users’ mobile phone numbers must be sought to join a group.

The positive predictors of SNSs uses identified by the included studies were younger age, fewer years of professional experience, and lower rank [36,44,48,50]. Other reviews on the use of SNSs in health communication and education also revealed that young people intend to use SNSs more than the older ones [11,51]. This observation aligns with the current profiles of SNS users such as 59% of active Facebook users are between the ages of 18 and 34 years [52]. It would warrant research exploring how this batch of “SNSs-competent” students would influence health communication when they come into practice in the near future.

In addition to the requirements on technical knowledge [28,29,36], the uncertainties on data protection and liability were also obstacles to the utilization of SNSs [28,29,43,44]. Moreover, the blurred border between the professional and personal spheres [39] and the risk of exposing one’s private life imposed further hesitation on using SNSs [22,29].

As the growth of SNSs is expected to rise, health professions should have a better understanding of how to attain secure and appropriate use of these platforms. Formal training should be provided to health professionals for the safe use of SNSs [33]. The American Medical Association recommends that physicians consider separating personal and professional information online, and they preserve professional boundaries when interacting with patients [53]. In a survey involving clerkship directors in the United States, most respondents felt that a faculty member accepting a friend request from a current student was never or rarely appropriate [54]. Yet, guidance on faculty-student or faculty-trainee interactions, particularly when SNSs are used as an educational tool, is often inadequate. Academic faculty could find it confusing to maintain appropriate boundaries in the instructor-student relationship [33].

Those concerns over the possible stigma and the negative impact of reputation on the use of SNSs fall within the context of e-professionalism. It is defined as the attitudes and behaviors that reflect traditional professionalism paradigms but are manifested through digital media [55,56]. E-professionalism is an essential and increasingly important element of professional identity formation [56]. Discussion on this topic helps to preserve the integrity of health professions, establish appropriate boundaries, and protect the privacy of both patients and professionals [57]. Unexpectedly, the relevant discussion was limited in most of the studies in this review. Evolving challenges are expected with the emerging use of SNSs; e-professionalism should be included in the education of health professionals and incorporated in institute policy and staff training.

Although not much mentioned in the included papers, a practical issue that should be given attention is how the SNS companies manage, analyze, repurpose, or even disclose the data and content of communication. According to the terms of service of Facebook, Twitter, WhatsApp Messenger, and LinkedIn, the companies reserve the right to collect, use, preserve, and share users’ information if it is deemed reasonably necessary to respond to legal process, government requests, or to enforce the companies’ terms and policies, and also under a list of other situations [58-61]. Health service institutes and providers must consider carefully in using SNSs for communicating confidential data to avoid jeopardizing patient privacy.

### Gaps in the Literature and Potential Areas for Further Research

In this review, the first key observation was the absence of an RCT among the included studies. All studies were exploratory in nature. The majority used surveys, content analysis, and thematic analysis. This illustrated the early phase of research in the field of professional use of SNSs when researchers were more concerned with describing health professionals’ behavior and opinions rather than the effectiveness of SNSs itself. The number of retrieved studies has risen considerably in the last 5 years, and it is expected to see significant growth in the research on SNSs soon. When this area of research advances further, research design will likely progress to interventional study. Some potential study designs are cross-sectional study, longitudinal study, and RCTs. It is worth mentioning that, nowadays, many analytics tools for SNSs are being developed in the market. Researchers can analyze straightforwardly how the content and performances of SNS interventions are affecting the study outcomes.

Every SNS is unique in design, interfaces, uses, and target users. To compare the relative effectiveness of SNSs for communication among health professionals, further research with more robust methodologies such as RCTs would be required. For instance, an RCT was conducted to investigate a physical activity intervention with pedometers delivered via Facebook app [62]. Another RCT was conducted to compare interventions via the WhatsApp Messenger and the Facebook social group in preventing smoking relapse in quitters [63].

With the emerging use of SNSs, evolving challenges in the context of e-professionalism are expected. This topic should be covered in the education of health professionals and incorporated in institute policy and staff training. Concerning the data policies of the SNSs companies, institutes must consider carefully in using SNSs for sharing confidential data. Research investigating the mechanisms of data protection and the potential risks in sharing information in SNSs should be conducted to identify suitable ways for safe use and maintenance of data.

Geographical locations may affect the generalization of findings in research on SNSs. The availability, acceptability, and popularity of SNSs vary across countries and populations. Twitter, among the top 3 SNSs in the United States, ranked ninth in Hong Kong, with only 10% of market share [64], whereas QZone, the top SNS in China, may not be heard by many Americans [8]. For higher applicability of findings to local practice, research has to be done in the corresponding location and jurisdiction. If published data are inadequate, exploratory study designs such as cross-sectional survey, preferably together with a validation study, should be conducted to explore health professionals’ perceptions, the barriers, and usage patterns of SNSs in professional communication. This helps to pave the way for research on more robust methodologies.

An effective and sustainable online network is crucial for the communication via SNSs. As discussed in the reviewed studies, not all the implementations of SNSs were found to be beneficial or effective [25,26,39]. Research could be done to explore strategies for designing and enhancing the usability of SNSs in communication among health professionals.

### Limitations of the Review

The absence of RCT coupled with the diverse and heterogeneous designs of the included studies has made conducting a meta-analysis unfeasible. Most studies were surveys and interviews, and their measurements and findings were mostly descriptive and qualitative. In addition, the questionnaires adopted in the surveys were mostly developed by the researchers, where validation might not be done. There were often some questions in common, such as asking respondents to distinguish the use of SNSs for personal or for professional purposes. Yet, the definitions of personal versus professional use of SNSs varied across studies. Without a well-stated explanation of terms, questions were sometimes ambiguous, for example, “How have you used or benefited from social media professionally?” [28].

The definitions of social media and SNSs could be unclear in some earlier literature. This complicates the analysis of the primary studies in this review. Another related limitation was the keyword search. The term “social network site” has not been added to the MeSH list in PubMed. This issue was addressed by undertaking a series of searches using a range of keywords, such as the names of common SNSs. Nevertheless, the searches may not have captured all relevant publications. Research on SNSs is growing so fast that evidence may have been published in electronic media or platforms not indexed through the academic databases. Thus, findings in this review are limited to research published in traditional peer-reviewed journals only.

### Conclusions

SNSs provide platforms facilitating efficient communication among health professionals in frontline clinical practice, professional networks, and education and training. Disseminating information, expanding professional connections, and promoting interactions are the benefits observed. Yet, the advantages come with limitations such as requirements on technical knowledge, professionalism issues, and risks of data protection. The evolving use of SNSs necessitates further robust research to explore the full potential and relative effectiveness of SNSs in professional communication.

## Appendix

Multimedia Appendix 1 Summary of the reviewed studies.

Multimedia Appendix 2 List of the excluded studies and the reasons for exclusion.

Multimedia Appendix 3 Quality of the reviewed studies.

## Abbreviations

CASP: Critical Appraisal Skills Programme
MeSH: medical subject heading
PRISMA: Preferred Reporting Items for Systematic Reviews and Meta-analyses
RCT: randomized clinical trial
SNS: social network site

## References

[1] Boyd DM, Ellison NB (2007). Social network sites: definition, history, and scholarship. J Comput Mediat Commun.

[2] Obar JA, Wildman S (2015). Social media definition and the governance challenge: an introduction to the special issue. Telecomm Policy.

[3] (2017). Statista.

[4] Facebook.

[5] (2017). Twitter.

[6] (2017). LinkedIn.

[7] WhatsApp Messenger (2017). Whatsapp.

[8] (2017). World Economic Forum.

[9] Capurro D, Cole K, Echavarría MI, Joe J, Neogi T, Turner AM (2014). The use of social networking sites for public health practice and research: a systematic review. J Med Internet Res.

[10] Moorhead SA, Hazlett DE, Harrison L, Carroll JK, Irwin A, Hoving C (2013). A new dimension of health care: systematic review of the uses, benefits, and limitations of social media for health communication. J Med Internet Res.

[11] Laranjo L, Arguel A, Neves AL, Gallagher AM, Kaplan R, Mortimer N, Mendes GA, Lau AY (2015). The influence of social networking sites on health behavior change: a systematic review and meta-analysis. J Am Med Inform Assoc.

[12] Rolls K, Hansen M, Jackson D, Elliott D (2016). How health care professionals use social media to create virtual communities: an integrative review. J Med Internet Res.

[13] Coiera E (2013). Social networks, social media, and social diseases. Br Med J.

[14] Moher D, Liberati A, Tetzlaff J, Altman DG (2009). Preferred reporting items for systematic reviews and meta-analyses: the PRISMA statement. Br Med J.

[15] (2017). Critical Appraisal Skills Programme.

[16] (2011). National Collaborating Centre for Methods & Tools.

[17] (2017). National Heart, Lung, and Blood Institute.

[18] Gruzd A, Haythornthwaite C (2013). Enabling community through social media. J Med Internet Res.

[19] Winandy M, Kostkova P, de Quincey E, St Louis C, Szomszor M (2016). Follow #eHealth2011: measuring the role and effectiveness of online and social media in increasing the outreach of a scientific conference. J Med Internet Res.

[20] Desselle SP (2017). The use of Twitter to facilitate engagement and reflection in a constructionist learning environment. Curr Pharm Teach Learn.

[21] Kostka-Rokosz MD, Camiel LD, McCloskey WW (2014). Pharmacy students' perception of the impact of a Facebook-delivered health news service-Two-year analysis. Curr Pharm Teach Learn.

[22] Mawdsley A, Schafheutle EI (2015). Using Facebook to support learning and exam preparation in a final-year undergraduate pharmacy clinical therapeutics module. Curr Pharm Teach Learn.

[23] Morley DA (2014). Supporting student nurses in practice with additional online communication tools. Nurse Educ Pract.

[24] Raiman L, Antbring R, Mahmood A (2017). WhatsApp messenger as a tool to supplement medical education for medical students on clinical attachment. BMC Med Educ.

[25] Reames BN, Sheetz KH, Englesbe MJ, Waits SA (2016). Evaluating the use of Twitter to enhance the educational experience of a medical school surgery clerkship. J Surg Educ.

[26] Wang T, Wang F, Shi L (2013). The use of microblog-based case studies in a pharmacotherapy introduction class in China. BMC Med Educ.

[27] Ganasegeran K, Renganathan P, Rashid A, Al-Dubai SA (2017). The m-Health revolution: exploring perceived benefits of WhatsApp use in clinical practice. Int J Med Inform.

[28] Patel SS, Hawkins CM, Rawson JV, Hoang JK (2017). Professional social networking in radiology: who is there and what are they doing?. Acad Radiol.

[29] Nikiphorou E, Studenic P, Ammitzbøll CG, Canavan M, Jani M, Ospelt C, Berenbaum F, EMEUNET (2017). Social media use among young rheumatologists and basic scientists: results of an international survey by the Emerging EULAR Network (EMEUNET). Ann Rheum Dis.

[30] Siegal G, Dagan E, Wolf M, Duvdevani S, Alon EE (2016). Medical information exchange: pattern of global mobile messenger usage among otolaryngologists. Otolaryngol Head Neck Surg.

[31] Fuoco M, Leveridge MJ (2015). Early adopters or laggards? Attitudes toward and use of social media among urologists. BJU Int.

[32] Narayanaswami P, Gronseth G, Dubinsky R, Penfold-Murray R, Cox J, Bever C, Martins Y, Rheaume C, Shouse D, Getchius TS (2015). The impact of social media on dissemination and implementation of clinical practice guidelines: a longitudinal observational study. J Med Internet Res.

[33] Cain J, Scott DR, Tiemeier AM, Akers P, Metzger AH (2013). Social media use by pharmacy faculty: student friending, e-professionalism, and professional use. Curr Pharm Teach Learn.

[34] Deen SR, Withers A, Hellerstein DJ (2013). Mental health practitioners' use and attitudes regarding the Internet and social media. J Psychiatr Pract.

[35] Keller B, Labrique A, Jain KM, Pekosz A, Levine O (2014). Mind the gap: social media engagement by public health researchers. J Med Internet Res.

[36] Stevens RJ, Hamilton NM, O’Donoghue JM, Davies MP (2012). The use of the Internet and social software by plastic surgeons. Eur J Plast Surg.

[37] Benetoli A, Chen TF, Schaefer M, Chaar BB, Aslani P (2016). Professional use of social media by pharmacists: a qualitative study. J Med Internet Res.

[38] Gulacti U, Lok U, Hatipoglu S, Polat H (2016). An analysis of WhatsApp usage for communication between consulting and emergency physicians. J Med Syst.

[39] Maisonneuve H, Chambe J, Lorenzo M, Pelaccia T (2015). How do general practice residents use social networking sites in asynchronous distance learning?. BMC Med Educ.

[40] Dieleman C, Duncan EA (2013). Investigating the purpose of an online discussion group for health professionals: a case example from forensic occupational therapy. BMC Health Serv Res.

[41] Flynn S, Hebert P, Korenstein D, Ryan M, Jordan WB, Keyhani S (2017). Leveraging social media to promote evidence-based continuing medical education. PLoS One.

[42] Goff DA, Jones C, Toney B, Nwomeh BC, Bauer K, Ellison EC (2016). Use of Twitter to educate and engage surgeons in infectious diseases and antimicrobial stewardship. Infect Dis Clin Pract.

[43] Lofters AK, Slater MB, Nicholas Angl E, Leung FH (2016). Facebook as a tool for communication, collaboration, and informal knowledge exchange among members of a multisite family health team. J Multidiscip Healthc.

[44] Barry AR, Pearson GJ (2015). Professional use of social media by pharmacists. Can J Hosp Pharm.

[45] Dong C, Cheema M, Samarasekera D, Rajaratnam V (2015). Using LinkedIn for continuing community of practice among hand surgeons worldwide. J Contin Educ Health Prof.

[46] Johnston MJ, King D, Arora S, Behar N, Athanasiou T, Sevdalis N, Darzi A (2015). Smartphones let surgeons know WhatsApp: an analysis of communication in emergency surgical teams. Am J Surg.

[47] Lipp A, Davis RE, Peter R, Davies JS (2014). The use of social media among health care professionals within an online postgraduate diabetes diploma course. Pract Diabetes.

[48] Loeb S, Bayne CE, Frey C, Davies BJ, Averch TD, Woo HH, Stork B, Cooperberg MR, Eggener SE, American Urological Association Social Media Work Group (2014). Use of social media in urology: data from the American Urological Association (AUA). BJU Int.

[49] Wani SA, Rabah SM, Alfadil S, Dewanjee N, Najmi Y (2013). Efficacy of communication amongst staff members at plastic and reconstructive surgery section using smartphone and mobile WhatsApp. Indian J Plast Surg.

[50] Wang AT, Sandhu NP, Wittich CM, Mandrekar JN, Beckman TJ (2012). Using social media to improve continuing medical education: a survey of course participants. Mayo Clin Proc.

[51] Benetoli A, Chen TF, Aslani P (2015). The use of social media in pharmacy practice and education. Res Social Adm Pharm.

[52] (2017). Statista.

[53] American Medical Association AMA.

[54] Chretien KC, Farnan JM, Greysen SR, Kind T (2011). To friend or not to friend? Social networking and faculty perceptions of online professionalism. Acad Med.

[55] Cain J, Romanelli F (2009). E-professionalism: a new paradigm for a digital age. Curr Pharm Teach Learn.

[56] Kaczmarczyk JM, Chuang A, Dugoff L, Abbott JF, Cullimore AJ, Dalrymple J, Davis KR, Hueppchen NA, Katz NT, Nuthalapaty FS, Pradhan A, Wolf A, Casey PM (2013). e-Professionalism: a new frontier in medical education. Teach Learn Med.

[57] Spector ND, Matz PS, Levine LJ, Gargiulo KA, McDonald MB, McGregor RS (2010). e-Professionalism: challenges in the age of information. J Pediatr.

[58] LinkedIn Corporation (2017). Linkedin.

[59] (2017). Twitter.

[60] WhatsApp Messenger (2017). Whatsapp.

[61] (2017). Facebook.

[62] Maher C, Ferguson M, Vandelanotte C, Plotnikoff R, De Bourdeaudhuij I, Thomas S, Nelson-Field K, Olds T (2015). A web-based, social networking physical activity intervention for insufficiently active adults delivered via Facebook app: randomized controlled trial. J Med Internet Res.

[63] Cheung YT, Chan CH, Lai CK, Chan WF, Wang MP, Li HC, Chan SS, Lam TH (2015). Using WhatsApp and Facebook online social groups for smoking relapse prevention for recent quitters: a pilot pragmatic cluster randomized controlled trial. J Med Internet Res.

[64] (2015). Go-globe.

