# Supplementary material for: Use of Social Network Sites for Communication Among Health Professionals: Systematic Review
Source: J Med Internet Res. 2018 Mar 28;20(3):e117. doi: 10.2196/jmir.8382 (PMC5895921; doi:10.2196/jmir.8382)
Supplement: Multimedia Appendix 1 [file jmir_v20i3e117_app1.pdf]

Multimedia Appendix 1: Summary of the reviewed studies (n=33).

Study (Year)	Study Objective	Study Design	Data Analysis Method	Samples/ Participant	Type of SNS	Conclusion/ Recommendations
Desselle (2017) [1]	To determine students' self-reported use of Twitter in a health systems course, gauge their perceptions of its value and utility for self-guided supplementation of course material, and evaluate the quality of students' reflections from information they found on Twitter	Cross-sectional survey (quantitative)	Descriptive analysis	67 pharmacy students	Twitter	The Twitter assignment was useful in students' learning. Students reported high levels of engagement in a course that previously had not been evaluated very highly.
Flynn et al. (2017) [2]	To examine the effectiveness of social media in engaging physicians in non-industry-sponsored continuing medical education	Mixed methods (quantitative and qualitative)	i. Modelling effects of interventions on clicks to a single landing site<br><br>ii. Thematic analysis on interviews	i. 592 clicks to the landing site<br><br>ii. 10 Physicians	Facebook and Twitter	Among the different modalities tested, Facebook led to the highest click-through rate.
Ganasegeran, Renganathan, Rashid, & Al-Dubai (2017) [3]	To investigate perceived benefits, if any, of WhatsApp use across general medical and emergency teams during clinical practice in Malaysia	Cross-sectional Survey (quantitative)	Descriptive analysis	307 medical and emergency department staff (nurses, medical assistants, medical residents, medical officers and physicians)	WhatsApp Messenger	WhatsApp was beneficial in clinical practice. Perceived benefits were significantly associated with usage characteristics and types of communication events.
Patel, Hawkins, Rawson, & Hoang (2017) [4]	To compare radiologists who use social networking for professional purposes to those who do not with regard to their characteristics, habits, and attitudes.	Cross-sectional survey (quantitative)	Descriptive analysis	186 radiologists	Any types of social media	Radiology is likely to see growth in the number of users and in the role of social networking in the coming years, as nearly half of professional users are radiology trainees.
Raiman, Antbring, & Mahmood (2017) [5]	To demonstrate the feasibility and acceptability of instant messaging communication to supplement medical education for medical students while on clinical	Mixed methods (qualitative)	Thematic analyses on i. content of WhatsApp messages and ii. structured	i. 582 messages ii. 19 third year medical students	WhatsApp Messenger	The results indicate the utility, feasibility and acceptability of WhatsApp Messenger in supplementing

	attachment		interviews			'problem-based learning' teaching.
Benetoli, Chen, Schaefer, Chaar, & Aslani (2016)<br>[6]	To investigate the professional use of social media by pharmacists	Exploratory qualitative study (qualitative)	Thematic analysis on the semi-structured interviews	31 pharmacists	Any types of social media	Participants used social media in a professional capacity, specifically for accessing and sharing health and professional information among peers.
Goff et al. (2016)<br>[7]	To evaluate Twitter as a tool to<br>engage and educate surgeons in emerging infectious disease (ID) topics on antibiotic resistance and antimicrobial stewardship	Mixed methods (qualitative and quantitative)	i. Content analysis of tweets<br><br>ii. Descriptive analysis of survey	i. 5117 tweets<br><br>ii. 21 Surgeons	Twitter	Twitter engaged surgeons in ID and antimicrobial stewardship topics and provided real-time education around antimicrobial resistance.
Gulacti, Lok, Hatipoglu, & Polat (2016)<br>[8]	To evaluate WhatsApp Messenger usage for communication between consulting and emergency physicians.	Retrospective observational study (qualitative)	Content analysis of WhatsApp messages	519 consultations requested by physicians	WhatsApp Messenger	WhatsApp is a useful communication tool between physicians, especially for emergency department consultants who are outside the hospital, because of the ability to transfer large amounts of clinical and radiological data during a short period of time.
Lofters, Slater, Angl, & Leung (2016)<br>[9]	To implement and evaluate a private Facebook group for members of a large Ontario multisite Family Health Team (FHT) to facilitate improved communication and collaboration.	Participatory action research (mixed methods, qualitative and quantitative)	i. Content analysis of Facebook posts<br>ii. descriptive analysis of survey	26 physicians and some nurses, dieticians, psychologists and other health professionals	Facebook	The implementation of a private Facebook group for a large, multisite FHT was ultimately not successful.
Nikiphorou et al. (2016)<br>[10]	To explore perceptions, barriers and patterns of social media use among rheumatology fellows and basic scientists.	Cross-sectional survey (quantitative)	Descriptive analysis	233 rheumatologists	Any types of social media	There was a substantial use of social media by rheumatologists and basic scientists for social and professional reasons. The survey highlights a need to provide learning resources and increase awareness of the use of social media.
Reames, Sheetz, Englesbe, &	To evaluate the use of Twitter as a novel educational tool in a medical school surgery	Prospective observational study	i. Descriptive analysis of survey	61 third-year medical students	Twitter	Applications such as Twitter can be facile educational tools to

Waits (2016)<br>[11]	clerkship.	(mixed methods, quantitative)	ii. Statistical analysis of aggregate test scores			supplement and enhance the experience of students on a medical school clerkship.
Siegal, Dagan, Wolf, Duvdevani, & Alon (2016)<br>[12]	To present the experience and practices governing the usage of WhatsApp, including data protection and privacy, of a national cohort of practicing otolaryngologists in Israel.	Cross-sectional survey (quantitative)	Descriptive analysis	69 otolaryngology	WhatsApp Messenger	The need to incorporate personal mobile devices in the overall information technology standards, guidelines, and regulation is becoming more acute.
Winandy, Kostkova, de Quincey, St Louis, & Szomszor (2016)<br>[13]	To propose a framework that defines and analyses the impact, outreach, and effectiveness of social media for event promotion and research dissemination to participants of a scientific event, as well as to a virtual audience through the Web.	Mixed methods (qualitative and quantitative)	i. Content analysis of communication<br>ii. Descriptive analysis of 5 longitudinal surveys	Targets of the eHealth 2011 conference	Twitter, Facebook, Flickr, and a Liveblog (with hashtag on Twitter)	A mix of Twitter, email, and a website can be recommended to achieve<br>the highest outreach before the conference, and these channels can be extended with Facebook and a Liveblog during the event, whereas the best channels after the event were Twitter and Flickr and proceedings.
Barry & Pearson (2015)<br>[14]	To characterize the use of social media by pharmacists in the Canadian province of Alberta and to identify independent determinants of and perceived barriers to using social media for professional purposes.	Mixed methods (quantitative and qualitative)	i. Descriptive analysis of survey<br>ii. Thematic analysis of focus group	i. 273 pharmacists<br>ii. 3 pharmacists	Any types of social media	Individuals and organizations seeking to expand their professional social media presence should focus on Twitter.
Dong, Cheema, Samarasekera, & Rajaratnam (2015)<br>[15]	To explore users' perception of the online community of practice in LinkedIn, and to identify features to aid the design and facilitation of similar online communities.	Participatory action research (mixed methods, quantitative and qualitative)	i. Descriptive analysis of survey<br><br>ii. Content analysis of user data	4106 Surgeons	LinkedIn	LinkedIn can serve as an effective online community of practice for hand surgeons to share knowledge and best practices.
Fuoco & Leveridge (2015)<br>[16]	To understand the attitudes and practices of urologists regarding social media use.	Cross-sectional survey (quantitative)	Descriptive analysis	229 urologists	Any types of social media	Practicing urologists engage infrequently in social media activities, and are almost universal in avoiding social media for professional use. Most feel that social media is best kept to

						exchanges between colleagues.
Johnston et al. (2015) [17]	To evaluate implementation of WhatsApp messaging service within emergency surgical teams.	Mixed methods (qualitative and quantitative)	i. Content analysis of communication events<br><br>ii. Thematic analysis of semi-structured interviews	i. 1495 communication events<br><br>ii. 40 emergency surgery team members	WhatsApp Messenger	The WhatsApp platform was deemed to be user-friendly and was extensively used to facilitate communication within a team.
Mawdsley & Schafheutle (2015) [18]	To gauge student opinion on teaching using social media	Exploratory participatory design (mixed methods, quantitative and qualitative)	i. Descriptive analysis of survey<br><br>ii. Content analysis of posts	i. 48 pharmacy students<br><br>ii. 142 active followers	Facebook	This evaluation demonstrates that students engage in social media learning, particularly if it is perceived as having a direct benefit to assessment.
Narayanaswami et al. (2015) [19]	To (1) develop an innovative dissemination strategy by adding social media-based dissemination methods to traditional methods for the AAN clinical practice guidelines “Complementary and alternative medicine (CAM) in multiple sclerosis” and (2) evaluate whether the addition of social media outreach improves awareness of the guideline.	Longitudinal, observational study (quantitative)	Descriptive analysis of survey	622 physicians	YouTube, Facebook, Twitter, LinkedIn	Social media-based dissemination methods did not confer additional benefits over print-, email-, and Internet-based methods in increasing guideline awareness and changing intent in physicians or patients.
Maisonneuve, Chambe, Lorenzo, & Pelaccia (2015) [20]	To explore the use of a social network site for asynchronous distance learning in a blended learning environment, as well as its influence on learners’ face-to-face interactions.	Prospective cohort study (qualitative)	Thematic analysis of semi-structured interviews	8 general practitioner residents	Any types of social media	Most of the general practice residentshad a positive appraisal on their use of SNS. We reported a positive impact on their engagement inlearning and their participation in discussions during face-to-face instruction.
Loeb et al. (2014) [21]	To characterize the use of social media among members of the American Urological Association.	Mixed methods (quantitative and qualitative)	i. Descriptive analysis of survey<br><br>ii. Content analysis on Twitter data	i. 382 urologists<br><br>ii. 5058 tweets	Any types of social media	Most urologists and urology trainees used some forms of social media, and its use in urology conferences has greatly expanded.

Lipp, Davis, Peter, & Davies (2014) [22]	To assess the impact of YouTube and Twitter among a group of health care professionals studying for a diploma in diabetes.	Participatory action research (mixed methods, quantitative and qualitative)	i. Descriptive analysis of survey<br><br>ii. Content analysis of media content and usage	89 HCP (nurse, physicians, pharmacists, dietitian…)	Twitter	Health professionals from a diverse background were able to adopt and effectively utilise social media platforms such as Twitter and YouTube to deliver health care messages.
Kostka-Rokosz, Camiel, & McCloskey (2014) [23]	To evaluate via a survey second-year Doctor of Pharmacy students' attitudes and perceptions of the impact of Facebook-delivered health care news.	Cross-sectional survey (quantitative)	Descriptive analysis	551 pharmacy students	Facebook	This project exposed students to an educational application of Facebook and encouraged them to explore and engage with social media as a way to facilitate their early professional development.
Keller, Labrique, Jain, Pekosz, & Levine (2014) [24]	To evaluate the extent to which public health professionals are engaged in social media	Cross-sectional survey (quantitative)	Descriptive analysis	181 faculty in public health	Any types of social media	A small minority are actually engaged in social media professionally, whereas most are either disinterested or actively opposed to professional engagement. Social media is seen by most as more useful for spreading information than obtaining it.
Morley (2014) [25]	To determine the usage of additional online communication support mechanisms by student nurses undertaking their first five-week clinical placement	Mixed methods (qualitative and quantitative)	i. Content analysis of communicatio n<br><br>ii. Descriptive analysis of survey	52 student nurses	Facebook and wiki group	Recommend using online communication tools already familiar to students to complement the support mechanisms that exist for practice learning.
Cain, Scott, Tiemeier, Akers, & Metzger (2013) [26]	(1) To examine social media use by pharmacy faculty members and their strategies for Facebook friending of students; and (2) To determine faculty members' opinions regarding e-professionalism, and their use of social media for teaching, learning, and professional interests.	Cross-sectional survey (quantitative)	Descriptive analysis	159 pharmacy faculty	Any types of social media	A majority of pharmacy faculty members have an online social media presence, with Facebook being the most common application.

Deen, Withers, & Hellerstein (2013) [27]	To better understand mental health providers' practices and attitudes regarding internet and social media.	Cross-sectional survey (quantitative)	Descriptive analysis	130 psychiatrists and psychologists	Any types of social media	Mental health care professionals are starting to incorporate Internet technologies into their professional lives, but they remain divided on the ethics and utility of using these technologies in clinical care.
Dieleman & Duncan (2013) [28]	To gain an understanding of the purpose and use of online discussion groups for health professionals who may be practically and geographically isolated from others in similar areas of practice	Case study design (qualitative)	Thematic analysis of communication in the discussion group	Forensic occupational therapists (no. not specified); 2494 posts	Yahoo online discussion group	Health professionals in specialized and often isolated areas of practice are keento connect with colleagues and learn from each other's experiences. Onlinediscussion groups could be used for communication, information sharing and networking.
Gruzd & Haythornthwaite (2013) [29]	To demonstrate how social network analysis provides a vocabulary and set of techniques for examining interaction patterns via social media.	Cross-sectional study (qualitative)	Content analysis and social network analysis	3871 tweets and 486 unique posters in the Health Care Social Media Canada (#hcsmca) Twitter community	Twitter	Network analysis and visualizations provide techniques and a vocabulary for understanding online interaction, as well as insights that can help in understanding what, and who, comprises and sustains a network, and whether community emerges from a network of online interactions.
Wang, Wang, & Shi (2013) [30]	To determine students' attitudes toward microblog-based case studies (MBC) in a pharmacotherapy class	Participatory action research (mixed methods, quantitative and qualitative)	i. Descriptive analysis of survey<br><br>ii. Content analysis of messages	i. 112 pharmacy students<br><br>ii. 592 messages	Sina Weibo	MBC appears to be a well-accepted learning method for students in this study.
Wani, Rabah, Alfadil, Dewanjee, & Najmi (2013) [31]	To assess the efficacy of smartphones and the WhatsApp application as a communication method among the staff of plastic and reconstructive surgery section at a tertiary care health	Prospective cohort study (mixed methods, quantitative and qualitative)	i. Descriptive analysis of survey<br><br>ii. Content analysis of messages	i. 40 plastic surgeons<br><br>ii. 116 episodes	WhatsApp Messenger	This new method of communication is an effective method for clinical and academic endorsements. The method is cheap and quick and easy to

	facility.					operate.
Stevens, Hamilton, O'Donoghue, & Davies (2012) [32]	To survey members of BAPRAS on their knowledge and use of Web 2.0 technology, and whether they would like this technology to be used in e-learning.	Cross-sectional survey (quantitative)	Descriptive analysis	58 plastic surgeons	Web 2.0	Most plastic surgeons either used Web 2.0 technology or were aware of it.
Wang et al (2012)[33]	To conduct a cross-sectional survey of US physicians attending a Mayo Clinic Internal Medicine continuous medical education (CME) course to determine their use of social media (SM) and evaluate their attitudes regarding the value of SM for enhancing CME	Cross-sectional survey (quantitative)	Descriptive analysis	327 Participants of the Mayo School of Continuous Professional Development	Any types of social media	The most fruitful categories of SM for CME use and marketing may be Facebook, YouTube, and Skype. The identified association between positive attitudes on using SM in CME with younger age and increased frequency of SM use suggests that CME course directors might want to direct SM learning strategies toward more youthful, technology-savvy CME physicians.

References:

1. Desselle SP. The use of Twitter to facilitate engagement and reflection in a constructionist learning environment. Currents in Pharmacy Teaching and Learning. 2017;9(2):185-94. doi: 10.1016/j.cptl.2016.11.016.
2. Flynn S, Hebert P, Korenstein D, Ryan M, Jordan WB, Keyhani S. Leveraging social media to promote evidence-based continuing medical education. PLoS ONE. 2017;12(1). doi: 10.1371/journal.pone.0168962.
3. Ganasegeran K, Renganathan P, Rashid A, Al-Dubai SAR. The m-Health revolution: Exploring perceived benefits of WhatsApp use in clinical practice. International Journal of Medical Informatics. 2017;97:145-51. doi: 10.1016/j.ijmedinf.2016.10.013.
4. Patel SS, Hawkins CM, Rawson JV, Hoang JK. Professional Social Networking in Radiology: Who Is There and What Are They Doing? Academic Radiology. 2017. doi: 10.1016/j.acra.2016.09.026.
5. Raiman L, Antbring R, Mahmood A. WhatsApp messenger as a tool to supplement medical education for medical students on clinical attachment. BMC Medical Education. 2017;17(1):7. PMID: 28061777.
6. Benetoli A, Chen TF, Schaefer M, Chaar BB, Aslani P. Professional Use of Social Media by Pharmacists: A Qualitative Study. Journal of Medical Internet Research. 2016;18(9):e258. PMID: 27663570.
7. Goff DA, Jones C, Toney B, Nwomeh BC, Bauer K, Ellison EC. Use of Twitter to Educate and Engage Surgeons in Infectious Diseases and Antimicrobial Stewardship. Infectious Diseases in Clinical Practice. 2016;24(6):324-7. doi: 10.1097/IPC.0000000000000440.
8. Gulacti U, Lok U, Hatipoglu S, Polat H. An Analysis of WhatsApp Usage for Communication Between Consulting and Emergency Physicians. Journal of Medical Systems. 2016;40(6). doi: 10.1007/s10916-016-0483-8.
9. Lofters AK, Slater MB, Angl EN, Leung FH. Facebook as a tool for communication, collaboration, and informal knowledge exchange among members of a multisite family health team. Journal of Multidisciplinary Healthcare. 2016;9:29-34. doi: 10.2147/JMDH.S94676.
10. Nikiphorou E, Studenic P, Ammitzbøll CG, Canavan M, Jani M, Ospelt C, et al. Social media use among young rheumatologists and basic scientists: Results of an international survey by the Emerging EULAR Network (EMEUNET). Annals of the Rheumatic Diseases. 2016. doi: 10.1136/annrheumdis-2016-209718.
11. Reames BN, Sheetz KH, Englesbe MJ, Waits SA. Evaluating the Use of Twitter to Enhance the Educational Experience of a Medical School Surgery Clerkship. Journal of Surgical Education. 2016;73(1):73-8. doi: 10.1016/j.jsurg.2015.08.005.
12. Siegal G, Dagan E, Wolf M, Duvdevani S, Alon EE. Medical Information Exchange: Pattern of Global Mobile Messenger Usage among Otolaryngologists. Otolaryngology - Head and Neck Surgery (United States). 2016;155(5):753-7. doi: 10.1177/0194599816656178.

13. Winandy M, Kostkova P, de Quincey E, St Louis C, Szomszor M. Follow #eHealth2011: Measuring the Role and Effectiveness of Online and Social Media in Increasing the Outreach of a Scientific Conference. Journal of Medical Internet Research. 2016;18(7):e191. PMID: 27436012.
14. Barry AR, Pearson GJ. Professional use of social media by pharmacists. Canadian Journal of Hospital Pharmacy. 2015;68(1):22-7. PMID: 25762816.
15. Dong C, Cheema M, Samarasekera D, Rajaratnam V. Using LinkedIn for Continuing Community of Practice Among Hand Surgeons Worldwide. Journal of Continuing Education in the Health Professions. 2015;35(3):185-91. PMID: 26378424.
16. Fuoco M, Leveridge MJ. Early adopters or laggards? Attitudes toward and use of social media among urologists. BJU International. 2015;115(3):491-7. PMID: 24981237.
17. Johnston MJ, King D, Arora S, Behar N, Athanasiou T, Sevdalis N, et al. Smartphones let surgeons know WhatsApp: an analysis of communication in emergency surgical teams. Am J Surg. 2015 Jan;209(1):45-51. PMID: 25454952. doi: 10.1016/j.amjsurg.2014.08.030.
18. Mawdsley A, Schafheutle EI. Using Facebook to support learning and exam preparation in a final-year undergraduate pharmacy clinical therapeutics module. Currents in Pharmacy Teaching and Learning. 2015;7(6):869-75. doi: 10.1016/j.cptl.2015.08.010.
19. Narayanaswami P, Gronseth G, Dubinsky R, Penfold-Murray R, Cox J, Bever C, Jr., et al. The Impact of Social Media on Dissemination and Implementation of Clinical Practice Guidelines: A Longitudinal Observational Study. Journal of Medical Internet Research. 2015;17(8):e193. PMID: 26272267.
20. Maisonneuve H, Chambe J, Lorenzo M, Pelaccia T. How do general practice residents use social networking sites in asynchronous distance learning? BMC Medical Education. 2015;15:154. PMID: 26391989.
21. Loeb S, Bayne CE, Frey C, Davies BJ, Averch TD, Woo HH, et al. Use of social media in urology: data from the American Urological Association (AUA). BJU International. 2014;113(6):993-8. PMID: 24274744.
22. Lipp A, Davis RE, Peter R, Davies JS. The use of social media among health care professionals within an online postgraduate diabetes diploma course. Practical Diabetes. 2014;31(1):14-7a. doi: 10.1002/pdi.1821.
23. Kostka-Rokosz MD, Camiel LD, McCloskey WW. Pharmacy students' perception of the impact of a Facebook-delivered health news service – a two-year analysis. Currents in Pharmacy Teaching and Learning. 2014;6(4):471-7. doi: 10.1016/j.cptl.2014.04.007.
24. Keller B, Labrique A, Jain KM, Pekosz A, Levine O. Mind the gap: social media engagement by public health researchers. Journal of Medical Internet Research. 2014;16(1):e8. PMID: 24425670.
25. Morley DA. Supporting student nurses in practice with additional online communication tools. Nurse Education in Practice. 2014;14(1):69-75. PMID: 104006548. Language: English. Entry Date: 20140128. Revision Date: 20150820. Publication Type: Journal Article. doi: 10.1016/j.nepr.2013.06.005.

26. Cain J, Scott DR, Tiemeier AM, Akers P, Metzger AH. Social media use by pharmacy faculty: Student friending, e-professionalism, and professional use. Currents in Pharmacy Teaching and Learning. 2013;5(1):2-8. doi: 10.1016/j.cptl.2012.09.002.
27. Deen SR, Withers A, Hellerstein DJ. Mental health practitioners' use and attitudes regarding the Internet and social media. Journal of Psychiatric Practice. 2013;19(6):454-63. PMID: 24241499.
28. Dieleman C, Duncan EA. Investigating the purpose of an online discussion group for health professionals: a case example from forensic occupational therapy. BMC Health Services Research. 2013;13:253. PMID: 23822895.
29. Gruzd A, Haythornthwaite C. Enabling community through social media. Journal of Medical Internet Research. 2013;15(10):e248. PMID: 24176835.
30. Wang T, Wang F, Shi L. The use of microblog-based case studies in a pharmacotherapy introduction class in China. BMC Medical Education. 2013;13:120. PMID: 24010945.
31. Wani SA, Rabah SM, Alfadil S, Dewanjee N, Najmi Y. Efficacy of communication amongst staff members at plastic and reconstructive surgery section using smartphone and mobile WhatsApp. Indian Journal of Plastic Surgery. 2013;46(3):502-5. PMID: 24459338.
32. Stevens RJG, Hamilton NM, O'Donoghue JM, Davies MP. The use of the Internet and social software by plastic surgeons. European Journal of Plastic Surgery. 2012;35(10):747-55. doi: 10.1007/s00238-011-0681-z.
33. Wang AT, Sandhu NP, Wittich CM, Mandrekar JN, Beckman TJ. Using social media to improve continuing medical education: a survey of course participants. Mayo Clin Proc. 2012 Dec;87(12):1162-70. PMID: 23141117. doi: 10.1016/j.mayocp.2012.07.024.
