# Supplementary material for: Use of Social Network Sites for Communication Among Health Professionals: Systematic Review
Source: J Med Internet Res. 2018 Mar 28;20(3):e117. doi: 10.2196/jmir.8382 (PMC5895921; doi:10.2196/jmir.8382)
Supplement: Multimedia Appendix 2 [file jmir_v20i3e117_app2.pdf]

Multimedia Appendix 2: List of the excluded studies and the reasons for exclusion

Excluded articles (n=177)

(1) Was not in English (n = 1)

- [1]

(2) Were reviews, reports, abstracts only, letters, and commentaries (n = 35)

- [2-36]

(3) Focused primarily on the communication between public/ patients and health professionals or for personal uses (n = 91)

- [37-127]

(4) Described the use of SNSs primarily on marketing or advertising focus (n = 24)

- [128-151]

(5) Studied non-SNS types of social media (n = 22)

- [152-173]

(6) Full-text were not available (n = 4)

- [174-177]

Full List of Excluded Articles (n=177)

1. Navas-Aparicio MDC. Health strategy: Networking of health services as an alternative to reducing waiting lists for surgery. Revista Chilena de Cirugia. 2017;69(2):184-8. doi: 10.1016/j.rchic.2016.10.016.
2. Topf JM, Sparks MA, Phelan PJ, Shah N, Lerma EV, Graham-Brown MPM, et al. The Evolution of the Journal Club: From Osler to Twitter. American Journal of Kidney Diseases. 2017. doi: 10.1053/j.ajkd.2016.12.012.
3. Fehring KA, de Martino I, McLawhorn AS, Sculco PK. Social media: physicians-to-physicians education and communication. Current Reviews in Musculoskeletal Medicine. 2017:1-3. doi: 10.1007/s12178-017-9411-x.
4. White R, Hayes C, White S, Hodson FJ. Using social media to challenge unwarranted clinical variation in the treatment of chronic noncancer pain: The “Brainman” story. Journal of Pain Research. 2016;9:701-9. doi: 10.2147/JPR.S115814.
5. Mallick S. Inter-institutional collaborative networking in the intellectual property rights regime: Research in plant molecular biology in India. International Journal of Biotechnology. 2016;14(2):89-111. doi: 10.1504/IJBT.2016.077940.
6. Gouda P, Das D, Clark A, Ezekowitz J. The impact of social media on cardiovascular medicine: Insights into Twitter's sphere of influence. 2016. p. S281-S2.
7. Giorgio A. Social networks, apps and smartphones for telemedicine. International Journal of Medical Engineering and Informatics. 2016;8(3):183-95. doi: 10.1504/IJMEI.2016.077436.
8. Toseeb U, Inkster B. Online social networking sites and mental health research. Frontiers in Psychiatry. 2015;6(MAR). doi: 10.3389/fpsyt.2015.00036.
9. Power A. Twitter’s potential to enhance professional networking. British Journal of Midwifery. 2015;23(1):65-7. PMID: 103867018. Language: English. Entry Date: 20150102. Revision Date: 20150820. Publication Type: Journal Article.
10. Power A. LinkedIn: Facebook for professionals? British Journal of Midwifery. 2015;23(3):196-8. PMID: 103771412. Language: English. Entry Date: 20150310. Revision Date: 20150820. Publication Type: Journal Article.
11. Power A. Is Facebook an appropriate platform for professional discourse? British Journal of Midwifery. 2015;23(2):140-2. PMID: 103748862. Language: English. Entry Date: 20150209. Revision Date: 20150820. Publication Type: Journal Article.
12. Dion X. Using social networking sites (namely Facebook) in health visiting practice -- an account of five years experience. Community Practitioner. 2015;88(2):28-31. PMID: 103804493. Language: English. Entry Date: 20150602. Revision Date: 20150820. Publication Type: Journal Article.

13. Choo EK, Ranney ML, Chan TM, Trueger NS, Walsh AE, Tegtmeyer K, et al. Twitter as a tool for communication and knowledge exchange in academic medicine: A guide for skeptics and novices. Medical Teacher. 2015;37(5):411-6. PMID: 103798863. Language: English. Entry Date: 20150512. Revision Date: 20161024. Publication Type: Journal Article. Journal Subset: Biomedical. doi: 10.3109/0142159X.2014.993371.
14. Chan TM, Thoma B, Lin M. Creating, curating, and sharing online faculty development resources: the medical education in cases series experience. Academic Medicine. 2015;90(6):785-9. PMID: 25785678.
15. Bullock A, Webb K. Technology in postgraduate medical education: A dynamic influence on learning? Postgraduate Medical Journal. 2015;91(1081):646-50. doi: 10.1136/postgradmedj-2014-132809.
16. Baum N. Online Reputation Management for Urologists. Urology Practice. 2015;2(2):69-72. doi: 10.1016/j.urpr.2014.09.007.
17. Lui J, Anderson CA, Matthews P, Nierenhausen E, Schlegelmilch A. Knowledge translation strategies to improve the resources for rehabilitation counselors to employ best practices in the delivery of vocational rehabilitation services. Journal of Vocational Rehabilitation. 2014;41(2):137-45. doi: 10.3233/JVR-140706.
18. Litterman NK, Rhee M, Swinney DC, Ekins S. Collaboration for rare disease drug discovery research. F1000Research. 2014;3. doi: 10.12688/f1000research.5564.1.
19. Howerton Child RJ, Mentes JC, Pavlish C, Phillips LR. Using Facebook and participant information clips to recruit emergency nurses for research. Nurse Researcher. 2014;21(6):16-21. PMID: 103977553. Language: English. Entry Date: 20140729. Revision Date: 20150819. Publication Type: Journal Article. doi: 10.7748/nr.21.6.16.e1246.
20. West C, Verran D. Something to tweet about: incorporating social media into your nursing practice. Transplant Journal of Australasia. 2013;22(1):10-2. PMID: 104278346. Language: English. Entry Date: 20130424. Revision Date: 20150819. Publication Type: Journal Article.
21. Potts DC, Hohler AD. Neurologists and technology: The changing "facebook" of practice. Neurology: Clinical Practice. 2013;3(2):149-54. doi: 10.1212/CPJ.0b013e31828d9ee4.
22. Hogan NM, Sweeney KJ. Social networking and scientific communication: A paradoxical return to Mertonian roots? Journal of the American Society for Information Science & Technology. 2013;64(3):644-6. PMID: 104318290. Language: English. Entry Date: 20130225. Revision Date: 20150820. Publication Type: Journal Article. Journal Subset: Computer/Information Science. doi: 10.1002/asi.22842.

23. Forgie SE, Duff JP, Ross S. Twelve tips for using Twitter as a learning tool in medical education. Medical Teacher. 2013;35(1):8-14. PMID: 104015024. Language: English. Entry Date: 20140202. Revision Date: 20150710. Publication Type: Journal Article. doi: 10.3109/0142159X.2012.746448.
24. Davis KJ. Exploring Virtual PLCs: Professional development for the busy practitioner. Perspectives on School-Based Issues. 2013;14(2):28-32. PMID: 104203205. Language: English. Entry Date: 20130805. Revision Date: 20150819. Publication Type: Journal Article. Journal Subset: Allied Health.
25. Yarrow L. Becoming social media savvy: Using web 2.0 to enhance education. Topics in Clinical Nutrition. 2012;27(1):34-40. doi: 10.1097/TIN.0b013e31824622a7.
26. Yanning Y, Sinclair K, Penman M. Using social networking for professional development of occupational therapy throughout the People's Republic of China. WFOT Bulletin. 2012;66:47-9. PMID: 104441947. Language: English. Entry Date: 20121128. Revision Date: 20150818. Publication Type: Journal Article.
27. Wiener L, Crum C, Grady C, Merchant M. To friend or not to friend: The use of social media in clinical oncology. Journal of Oncology Practice. 2012;8(2):103-6. doi: 10.1200/JOP.2011.000357.
28. Stewart S, Sidebotham M, Davis D. International networking: connecting midwives through social media. International Nursing Review. 2012;59(3):431-4. PMID: 22897197.
29. Schmitt TL, Lilly K. Social media use among nurses. Journal of the Dermatology Nurses' Association. 2012;4(3):181-7.
30. Rogers L. Twitter-obsessed nurses lack the social skills of the older generation. Nursing Standard. 2012;26(32):32-. PMID: 104561725. Language: English. Entry Date: 20120511. Revision Date: 20150711. Publication Type: Journal Article.
31. Marsden D, Green A, March T, Ledington S. The role of technology in learning disability nursing. Learning Disability Practice. 2012;15(3):28-30. PMID: 104555839. Language: English. Entry Date: 20120502. Revision Date: 20150820. Publication Type: Journal Article.
32. Friesen N, Lowe S. The questionable promise of social media for education: connective learning and the commercial imperative. Journal of Computer Assisted Learning. 2012;28(3):183-94. PMID: 104568436. Language: English. Entry Date: 20120615. Revision Date: 20150819. Publication Type: Journal Article. doi: 10.1111/j.1365-2729.2011.00426.x.
33. Griffith R, Tengnah C. District nurses' use of social networking sites: caution required. British Journal of Community Nursing. 2011;16(9):455-7. PMID: 104636432. Language: English. Entry Date: 20111027. Revision Date: 20150820. Publication Type: Journal Article.

34. Van Dusen VR, Whatley NL. Electronic communications by pharmacy employees: Legal issues and practical concerns. Journal of Pharmacy Technology. 2010;26(1):9-13.
35. Thompson C. Facebook -- cautionary tales for nurses. Kai Tiaki Nursing New Zealand. 2010;16(7):26-. PMID: 105078736. Language: English. Entry Date: 20101001. Revision Date: 20150820. Publication Type: Journal Article. Journal Subset: Australia & New Zealand.
36. Hansen M, Erdley S. YouTube and other Web 2.0 applications for nursing education. Online Journal of Nursing Informatics. 2009;13(3):1-20. PMID: 105264451. Language: English. Entry Date: 20100212. Revision Date: 20150820. Publication Type: Journal Article. Journal Subset: Computer/Information Science.
37. Wong R, Harris JK, Staub M, Bernhardt JM. Local Health Departments Tweeting About Ebola: Characteristics and Messaging. Journal of Public Health Management & Practice. 2017;23(2):e16-e24. PMID: 26334537.
38. Sabato LA, Barone C, McKinney K. Use of social media to engage membership of a state health-system pharmacy organization. American Journal of Health-System Pharmacy. 2017;74(1):e72-e5. doi: 10.2146/ajhp151042.
39. Duymuş TM, Karadeniz H, Şükür E, Atiç R, Zehir S, Azboy İ. Social media and Internet usage of orthopaedic surgeons. Journal of Clinical Orthopaedics and Trauma. 2017;8(1):25-30. doi: 10.1016/j.jcot.2016.10.007.
40. Desselle SP. The use of Twitter to facilitate engagement and reflection in a constructionist learning environment. Currents in Pharmacy Teaching and Learning. 2017;9(2):185-94. doi: 10.1016/j.cptl.2016.11.016.
41. Borgmann H, Woelm J, Nelson K, Gust K, Mager R, Reiter M, et al. Strategy of robotic surgeons to exert public influence through Twitter. International Journal of Medical Robotics and Computer Assisted Surgery. 2017;13(1). doi: 10.1002/rcs.1739.
42. Benetoli A, Chen TF, Schaefer M, Chaar B, Aslani P. Do pharmacists use social media for patient care? International Journal of Clinical Pharmacy. 2017:1-9. doi: 10.1007/s11096-017-0444-4.
43. Walshe C, Dodd S, Hill M, Ockenden N, Payne S, Perez Algorta G, et al. Working with non-clinical staff to deliver research. Lessons from running a wait-list controlled trial (ELSA) of a volunteer befriending service towards the end of life. Palliative Medicine. 2016;30(6):NP283-NP4. doi: 10.1177/0269216316646056.
44. Waldrop J, Wink D. Twitter: An Application to Encourage Information Seeking Among Nursing Students. Nurse Educator. 2016;41(3):160-3. PMID: 26673316.
45. Udovicich C, Barberi A, Perera K. Tweeting the meeting: A comparative analysis of an Australian emergency medicine conference over four years. Journal of Emergencies, Trauma and Shock. 2016;9(1):28-31. doi: 10.4103/0974-2700.161655.

46. Stephens TM, Gunther ME. Twitter, Millennials, and Nursing Education Research. Nursing Education Perspectives. 2016;37(1):23-7. PMID: 27164773.
47. Sattar K, Ahmad T, Abdulghani HM, Khan S, John J, Meo SA. Social networking in medical schools: Medical student’s viewpoint. Biomedical Research (India). 2016;27(4):1378-84.
48. Salem J, Borgmann H, Bultitude M, Martin Fritsche H, Haferkamp A, Heidenreich A, et al. Online discussion on #KidneyStones: A longitudinal assessment of activity, users and content. PLoS ONE. 2016;11(8). doi: 10.1371/journal.pone.0160863.
49. McNair AGK, Brookes ST, Whistance RN, Forsythe RO, Macefield R, Rees J, et al. Trial outcomes and information for clinical decision-making: A comparative study of opinions of health professionals. Trials. 2016;17(1). doi: 10.1186/s13063-016-1492-0.
50. Lagu T, Goff SL, Craft B, Calcasola S, Benjamin EM, Priya A, et al. Can social media be used as a hospital quality improvement tool? Journal of Hospital Medicine. 2016;11(1):52-5. doi: 10.1002/jhm.2486.
51. Jayaram M, Adams C, Bodart A, Sampson S, Zhao S, Montgomery A. Tweeting links to cochrane schizophrenia group reviews: A randomised controlled trial. Australian and New Zealand Journal of Psychiatry. 2016;50:183. doi: 10.1177/0004867416640967.
52. Hawkins CM, Hunter M, Kolenic GE, Carlos RC. Social Media and Peer-Reviewed Medical Journal Readership: A Randomized Prospective Controlled Trial. Journal of the American College of Radiology. 2016. doi: 10.1016/j.jacr.2016.12.024.
53. Hand RK, Kenne D, Wolfram TM, Abram JK, Fleming M. Assessing the Viability of Social Media for Disseminating Evidence-Based Nutrition Practice Guideline Through Content Analysis of Twitter Messages and Health Professional Interviews: An Observational Study. Journal of Medical Internet Research. 2016;18(11):e295. PMID: 27847349.
54. Feret B, Romano S. Social media utilization among pharmacy preceptors. Currents in Pharmacy Teaching and Learning. 2016;8(4):555-8. doi: 10.1016/j.cptl.2016.03.004.
55. Ebrahimpour A, Rajabali F, Yazdanfar F, Azarbad R, Nodeh MR, Siamian H, et al. Social network sites as educational factors. Acta Informatica Medica. 2016;24(2):134-8. doi: 10.5455/aim.2016.24.134-138.
56. Diaz F, Gamon M, Hofman JM, Kiciman E, Rothschild D. Online and social media data as an imperfect continuous panel survey. PLoS ONE. 2016;11(1). doi: 10.1371/journal.pone.0145406.
57. Cardona-Grau D, Sorokin I, Leinwand G, Welliver C. Introducing the Twitter

Impact Factor: An Objective Measure of Urology's Academic Impact on Twitter. European Urology Focus. 2016;2(4):412-7. doi: 10.1016/j.euf.2016.03.006.
58. Campagne J, Galland J, Mangin O. Use of Twitter® in a congress: First experience for French internal medicine. Revue de Medecine Interne. 2016;37(7):497-501. doi: 10.1016/j.revmed.2015.10.340.
59. Borgmann H, Loeb S, Salem J, Thomas C, Haferkamp A, Murphy DG, et al. Activity, content, contributors, and influencers of the twitter discussion on urologic oncology. Urologic Oncology: Seminars and Original Investigations. 2016;34(9):377-83. doi: 10.1016/j.urolonc.2016.02.021.
60. Bombaci SP, Farr CM, Gallo HT, Mangan AM, Stinson LT, Kaushik M, et al. Using Twitter to communicate conservation science from a professional conference. Conservation Biology. 2016;30(1):216-25. PMID: 26081769.
61. Attai DJ, Radford DM, Cowher MS. Tweeting the Meeting: Twitter Use at The American Society of Breast Surgeons Annual Meeting 2013–2016. Annals of Surgical Oncology. 2016;23(10):3418-22. doi: 10.1245/s10434-016-5406-x.
62. Alpert JM, Womble FE. Just What the Doctor Tweeted: Physicians' Challenges and Rewards of Using Twitter. Health Communication. 2016;31(7):824-32. PMID: 26644165.
63. Young SWH, Rossmann D. Building Library Community Through Social Media. Information Technology & Libraries. 2015;34(1):20-37. PMID: 103786230. Language: English. Entry Date: 20150408. Revision Date: 20150820. Publication Type: Journal Article.
64. Wilkinson SE, Basto MY, Perovic G, Lawrentschuk N, Murphy DG. The social media revolution is changing the conference experience: analytics and trends from eight international meetings. BJU International. 2015;115(5):839-46. PMID: 25130687.
65. Powell G, Bell H, Rodriguez H, Thomas M, Painter J, Harvey C, et al. Comparison of events in spontaneous adverse event reports to events discussed within context of drug use on facebook and twitter. Pharmacoepidemiology and Drug Safety. 2015;24:441. doi: 10.1002/pds.3838.
66. Pan Y, Kang X, Luo H, Guo X. Instruction delivered by mobile social media app increases quality of bowel preparation and adenoma detection rate: A prospective, colonoscopist-blinded, randomised, controlled study. Gastrointestinal Endoscopy. 2015;81(5):AB146. doi: 10.1016/j.gie.2015.03.1233.
67. Ozutemiz C, Dicle O, Koremezli N. How Turkish radiology residents access information related to their profession in this social media and smartphone era. European Journal of Radiology Open. 2015;2:129-33. doi: 10.1016/j.ejro.2015.10.001.

68. Nakada H, Tsubokura M, Kishi Y, Yuji K, Matsumura T, Kami M. How do medical journalists treat cancer-related issues? ecancermedicalscience. 2015;9:1-6. doi: 10.3332/ecancer.2015.502.
69. Marnocha S, Marnocha MR, Pilliow T. Unprofessional content posted online among nursing students. Nurse Educator. 2015;40(3):119-23. PMID: 25501656.
70. Maher C, Ferguson M, Vandelanotte C, Plotnikoff R, De Bourdeaudfuji I, Thomas S, et al. An online social networking physical activity intervention delivered via Facebook: A randomised controlled trial. Journal of Science and Medicine in Sport. 2015;19:e10. doi: 10.1016/j.jsams.2015.12.404.
71. Loeb S, Bayne CE, Frey C, Davies BJ, Averch TD, Woo HH, et al. Updated Survey of Social Media Use by Members of the American Urological Association. Urology Practice. 2015;2(3):138-43. doi: 10.1016/j.urpr.2014.09.009.
72. Jawaid M, Khan MH, Bhutto SN. Social network utilization (Facebook)&e-Professionalism among medical students. Pakistan Journal of Medical Sciences. 2015;31(1):209-13. doi: 10.12669/pjms.311.5643.
73. Hu C-P, Yan W-W, Hu Y. User satisfaction evaluation of microblogging services in China: using the tetra-class model. Behaviour & Information Technology. 2015;34(1):17-32. PMID: 103862783. Language: English. Entry Date: 20141223. Revision Date: 20160624. Publication Type: Journal Article. doi: 10.1080/0144929X.2014.942753.
74. Devi TR, Arun K. Social networking for video sharing. Journal of Chemical and Pharmaceutical Sciences. 2015;2015-July:177-9.
75. Davies N, Murphy DG, van Rij S, Woo HH, Lawrentschuk N. Online and social media presence of Australian and New Zealand urologists. BJU International. 2015;116(6):984-9. PMID: 25906813.
76. Cox-George C. The changing face(book) of psychiatry: Can we justify 'following' patients' social media activity? Psychiatrist. 2015;39(6):283-4. doi: 10.1192/pb.bp.114.049130.
77. Banks CE, Randviir EP, Ilingworth SM, Baker MJ, Cude M. Twittering About Research: A Case Study of the World's First Twitter Poster Competition. F1000Research. 2015;4. doi: 10.12688/f1000research.6992.2.
78. Awad NI, Cocchio C. Use of Twitter at a major national pharmacy conference. American Journal of Health-System Pharmacy. 2015;72(1):65-9. PMID: 103752321. Language: English. Entry Date: 20150206. Revision Date: 20150710. Publication Type: Journal Article. doi: 10.2146/ajhp140175.
79. Augustine JM, Jackowski McKinley RM, Warholak TL, Yehoshua A, Ip Q, Armstrong EP. Perceptions of student pharmacists on professionalism and social networking sites: A Rasch analysis. Currents in Pharmacy Teaching and Learning.

2015;7(5):645-55. doi: 10.1016/j.cptl.2015.06.012.
80. Alkhateeb FM, Alameddine S, Attarabeen O, Latif DA, Osolin S, Khanfar N, et al. Pharmacy students' use of social media sites and perception toward Facebook use. Archives of Pharmacy Practice. 2015;6(4):77-84. doi: 10.4103/2045-080X.165134.
81. Al-Hariri MT, Al-Hattami AA. Utilization of internet by health colleges students at the University of Dammam. Journal of Taibah University Medical Sciences. 2015;10(1):66-73. doi: 10.1016/j.jtumed.2015.01.006.
82. Weber ZA, Vincent AH. Facebook as a method to promote a mindset of continual learning in an ambulatory care pharmacy elective course. Currents in Pharmacy Teaching and Learning. 2014;6(4):478-82. doi: 10.1016/j.cptl.2014.04.009.
83. Mishori R, Singh LO, Levy B, Newport C. Mapping physician Twitter networks: describing how they work as a first step in understanding connectivity, information flow, and message diffusion. Journal of Medical Internet Research. 2014;16(4):e107. PMID: 24733146.
84. Lenormand M, Picornell M, Cantú-Ros OG, Tugores A, Louail T, Herranz R, et al. Cross-checking different sources of mobility information. PLoS ONE. 2014;9(8). doi: 10.1371/journal.pone.0105184.
85. Kung YM, Oh S. Characteristics of nurses who use social media. CIN: Computers, Informatics, Nursing. 2014;32(2):64-72; quiz 3-4. PMID: 24419089.
86. Jain A, Petty EM, Jaber RM, Tackett S, Purkiss J, Fitzgerald J, et al. What is appropriate to post on social media? Ratings from students, faculty members and the public. Medical Education. 2014;48(2):157-69. PMID: 24528398.
87. Fu S, Huang J, Yan Y, Ou Y. Research on undergraduates’ continuous using behaviors of WeChat: Data from China. Journal of Chemical and Pharmaceutical Research. 2014;6(6):125-30.
88. Frazier B, Culley JM, Hein LC, Williams A, Tavakoli AS. Social networking policies in nursing education. CIN: Computers, Informatics, Nursing. 2014;32(3):110-7. PMID: 24406310.
89. Dvorkin Camiel L, Goldman-Levine JD, Kostka-Rokosz MD, McCloskey WW. Twitter as a medium for pharmacy students' personal learning network development. Currents in Pharmacy Teaching and Learning. 2014;6(4):463-70. doi: 10.1016/j.cptl.2014.04.008.
90. Curry E, Li X, Nguyen J, Matzkin E. Prevalence of Internet and social media usage in orthopedic surgery. Orthopedic Reviews. 2014;6(3):107-11. doi: 10.4081/or.2014.5483.
91. Cocchio C, Awad N. The scholarly merit of social media use among clinical faculty. Journal of Pharmacy Technology. 2014;30(2):61-8. doi: 10.1177/8755122513518497.

92. Bales ME, Dine DC, Merrill JA, Johnson SB, Bakken S, Weng C. Associating co-authorship patterns with publications in high-impact journals. Journal of Biomedical Informatics. 2014;52:311-8. doi: 10.1016/j.jbi.2014.07.015.
93. Bagley JE, Digiacinto D, Lawyer J, Anderson MP. Health care students who frequently use facebook are unaware of the risks for violating HIPAA standards: A pilot study. Journal of Diagnostic Medical Sonography. 2014;30(3):114-20. doi: 10.1177/8756479314530509.
94. Young SD, Cumberland WG, Lee SJ, Jaganath D, Szekeres G, Coates T. Social networking technologies as an emerging tool for HIV prevention. Annals of Internal Medicine. 2013;159(5):318-24. doi: 10.7326/0003-4819-159-5-201309030-00005.
95. White J, Kirwan P, Lai K, Walton J, Ross S. 'Have you seen what is on Facebook?' the use of social networking software by healthcare professions students. BMJ Open. 2013;3(7). doi: 10.1136/bmjopen-2013-003013.
96. Strausburg MB, Djuricich AM, Carlos WG, Bosslet GT. The influence of the residency application process on the online social networking behavior of medical students: a single institutional study. Academic Medicine. 2013;88(11):1707-12. PMID: 24072117.
97. Sato A, Costa-i-Font J. Social networking for medical information: A digital divide or a trust inquiry? Health Policy and Technology. 2013;2(3):139-50. doi: 10.1016/j.hlpt.2013.05.002.
98. Plumb AM. Student Perceptions of Social Networking as a Supplemental Learning Tool in the Communication Disorders Classroom. Contemporary Issues in Communication Science & Disorders. 2013 Fall2013;40:170-9. PMID: 104050940. Language: English. Entry Date: 20140318. Revision Date: 20150818. Publication Type: Journal Article.
99. Mbuagbaw L, Morfaw F, Kunda JEL, Mukonzo JK, Kastner J, Zhang S, et al. Tips for charting the course of a successful health research career. Journal of Multidisciplinary Healthcare. 2013;6:163-8. doi: 10.2147/JMDH.S44738.
100. Keogh K. Nurses warned of disciplinary risk over improper Facebook postings. Nursing Standard. 2013;27(52):7-. PMID: 104219716. Language: English. Entry Date: 20130903. Revision Date: 20150711. Publication Type: Journal Article.
101. Farooqi H, Patel H, Aslam HM, Ansari IQ, Khan M, Iqbal N, et al. Effect of Facebook on the life of Medical University students. International Archives of Medicine. 2013;6(1). doi: 10.1186/1755-7682-6-40.
102. David MA, Hamid Hashmi SS. Study to evaluate prevalence of depression, sleep wake pattern and their relation with use of social networking sites among first year medical students. International Journal of Pharma Medicine and Biological Sciences. 2013;2(1):27-31.

103. Brynolf A, Johansson S, Appelgren E, Lynoe N, Bonamy AKE. Virtual colleagues, virtually colleagues- Physicians' use of Twitter: A population-based observational study. BMJ Open. 2013;3(7). doi: 10.1136/bmjopen-2013-002988.
104. Allison MP. Use of Social Networking Sites in the Communication Disorders Classroom. Perspectives on Issues in Higher Education. 2013;16(1):17-27. PMID: 104205078. Language: English. Entry Date: 20130806. Revision Date: 20150819. Publication Type: Journal Article.
105. Ahmed O, Sullivan SJ, Schneiders A, Moon S, McCrory P. Exploring the opinions and perspectives of general practitioners towards the use of social networking sites for concussion management. Journal of Primary Health Care. 2013;5(1):36-42. PMID: 104245998. Language: English. Entry Date: 20130306. Revision Date: 20150711. Publication Type: Journal Article.
106. Witt CM, Holmberg C. Changing academic medicine: Strategies used by academic leaders of integrative medicine-A qualitative study. Evidence-based Complementary and Alternative Medicine. 2012;2012. doi: 10.1155/2012/652546.
107. Van de Belt TH, Berben SA, Samsom M, Engelen LJ, Schoonhoven L. Use of social media by Western European hospitals: longitudinal study. Journal of Medical Internet Research. 2012;14(3):e61. PMID: 22549016.
108. Stewart SA, Abidi SS. Applying social network analysis to understand the knowledge sharing behaviour of practitioners in a clinical online discussion forum. J Med Internet Res. 2012 Dec 04;14(6):e170. PMID: 23211783. doi: 10.2196/jmir.1982.
109. Peluchette J, Karl K, Coustasse A, Emmett D. Professionalism and social networking: can patients, physicians, nurses, and supervisors all be "friends?". Health Care Manager. 2012;31(4):285-94. PMID: 23111479.
110. McKendrick DR, Cumming GP, Lee AJ. Increased use of Twitter at a medical conference: a report and a review of the educational opportunities. Journal of Medical Internet Research. 2012;14(6):e176. PMID: 23232765.
111. Kamel Boulos MN, Anderson PF. Preliminary survey of leading general medicine journals' use of Facebook and Twitter. Journal of the Canadian Health Libraries Association (JCHLA). 2012;33(2):38-47. PMID: 104194381. Language: English. Entry Date: 20130722. Revision Date: 20150819. Publication Type: Journal Article.
112. Golden JB, Sweeny L, Bush B, Carroll WR. Social networking and professionalism in otolaryngology residency applicants. Laryngoscope. 2012;122(7):1493-6. PMID: 22689312.
113. Englund H, Chappy S, Jambunathan J, Gohdes E. Ethical reasoning and online social media. Nurse Educator. 2012;37(6):242-7. PMID: 23086065.

114. Chaudhry A, Glodé LM, Gillman M, Miller RS. Trends in twitter use by physicians at the American Society of Clinical Oncology Annual Meeting, 2010 and 2011. Journal of Oncology Practice. 2012;8(3):173-8. doi: 10.1200/JOP.2011.000483.
115. Thompson LA, Black E, Duff WP, Paradise Black N, Saliba H, Dawson K. Protected health information on social networking sites: ethical and legal considerations. Journal of Medical Internet Research. 2011;13(1):e8. PMID: 21247862.
116. Mistry V. Critical care training: using Twitter as a teaching tool. British Journal of Nursing. 2011;20(20):1292-6. PMID: 108216886. Language: English. Entry Date: 20120113. Revision Date: 20150820. Publication Type: Journal Article.
117. Glover CM, Frounfelker RL. Competencies of employment specialists for effective job development. American Journal of Psychiatric Rehabilitation. 2011;14(3):198-211. doi: 10.1080/15487768.2011.598093.
118. Chretien KC, Farnan JM, Greysen SR, Kind T. To friend or not to friend? Social networking and faculty perceptions of online professionalism. Academic Medicine. 2011;86(12):1545-50. PMID: 22030752.
119. Chilvers J. Implementation of a Facebook page by school nurses. Community Practitioner. 2011;84(4):33-5. PMID: 104851193. Language: English. Entry Date: 20110406. Revision Date: 20150820. Publication Type: Journal Article. Journal Subset: Blind Peer Reviewed.
120. MacDonald J, Sohn S, Ellis P. Privacy, professionalism and Facebook: a dilemma for young doctors. Medical Education. 2010;44(8):805-13. PMID: 20633220.
121. Finn G, Garner J, Sawdon M. 'You're judged all the time!' Students' views on professionalism: a multicentre study. Medical Education. 2010;44(8):814-25. PMID: 20633221.
122. Coffey M, Coufopoulos A. Creating a 'Health Promoting Curriculum' to inform the development of a Health Promoting University: A case study. International Journal of Health Promotion and Education. 2010;48(1):4-8.
123. Clauson KA, Elkins J, Goncz CE. Use of blogs by pharmacists. American Journal of Health-System Pharmacy. 2010;67(23):2043-8. PMID: 104956610. Language: English. Entry Date: 20101210. Revision Date: 20150711. Publication Type: Journal Article. doi: 10.2146/ajhp100065.
124. Aynsley S. Twittering for health. Libraries for Nursing Bulletin. 2010;30(2):12-8. PMID: 105050426. Language: English. Entry Date: 20100820. Revision Date: 20150818. Publication Type: Journal Article.
125. Graffeo I, La Barbera D. Cybertherapy meets facebook, blogger, and second life: An Italian experience. Annual Review of CyberTherapy and Telemedicine.

2009;7(1):108-12.
126. Schleyer T, Spallek H, Butler BS, Subramanian S, Weiss D, Poythress ML, et al. Facebook for scientists: requirements and services for optimizing how scientific collaborations are established. Journal of Medical Internet Research. 2008;10(3):e24. PMID: 18701421.
127. Cravens JD, Whiting JB. Clinical Implications of Internet Infidelity: Where Facebook Fits In. American Journal of Family Therapy. 2008;36(2):325-39. PMID: 103965629. Language: English. Entry Date: 20140711. Revision Date: 20150710. Publication Type: Journal Article. Journal Subset: Biomedical. doi: 10.1080/01926187.2013.874211.
128. Ng JP, Tarazi N, Byrne DP, Baker JF, McCabe JP. Scoliosis and the Social Media: Facebook as a Means of Information Exchange. Spine Deformity. 2017;5(2):102-8. doi: 10.1016/j.jspd.2016.11.003.
129. Yang YM, Jeong E, Je NK, Jee JP, Yoo JC, Choi EJ. An analysis of pharmacy students' social networking service activities and perceptions regarding e-professionalism under the newly implemented 6-year pharmacy educational system in South Korea. Indian Journal of Pharmaceutical Education and Research. 2016;50(1):63-9. doi: 10.5530/ijper.50.1.9.
130. Warner ET, Carapinha R, Weber GM, Hill EV, Reede JY. Faculty Promotion and Attrition: The Importance of Coauthor Network Reach at an Academic Medical Center. Journal of General Internal Medicine. 2016;31(1):60-7. doi: 10.1007/s11606-015-3463-7.
131. Sarode SC, Sarode GS, Anand R, Patil S, Unadkat H. WhatsApp is an effective tool for obtaining second opinion in oral pathology practice. Journal of Oral Pathology and Medicine. 2016. doi: 10.1111/jop.12515.
132. Lloyd GF, Singh S, Barclay P, Goh S, Bajorek B. Hospital pharmacists' perspectives on the role of key performance indicators in Australian pharmacy practice. Journal of Pharmacy Practice and Research. 2016. doi: 10.1002/jppr.1156.
133. Kelly BS, Redmond CE, Nason GJ, Healy GM, Horgan NA, Heffernan EJ. The Use of Twitter by Radiology Journals: An Analysis of Twitter Activity and Impact Factor. Journal of the American College of Radiology. 2016;13(11):1391-6. doi: 10.1016/j.jacr.2016.06.041.
134. Halboub E, Othathi F, Mutawwam F, Madkhali S, Somaili D, Alahmar N. Effect of social networking on academic achievement of dental students, Jazan University, Saudi Arabia. Eastern Mediterranean Health Journal. 2016;22(12):865-71.
135. Currie G, Woznitza N, Bolderston A, Westerink A, Watson J, Beardmore C, et al. Twitter Journal Club in Medical Radiation Science. Journal of Medical Imaging and Radiation Sciences. 2016. doi: 10.1016/j.jmir.2016.09.001.

136. Alotaibi NM, Badhiwala JH, Nassiri F, Guha D, Ibrahim GM, Shamji MF, et al. The Current Use of Social Media in Neurosurgery. World Neurosurgery. 2016;88:619-24. doi: 10.1016/j.wneu.2015.11.011.
137. Weiler MI, Santanello CD, Isaacs D, Rahman A, O'Donnell EP, Peters GL. Pharmacy students' attitudes about social media use at five schools of pharmacy. Currents in Pharmacy Teaching and Learning. 2015;7(6):804-10. doi: 10.1016/j.cptl.2015.08.013.
138. Robinson L, Behi O, Corcoran A, Cowley V, Cullinane J, Martin I, et al. Evaluation of whatsapp for promoting social presence in a first year undergraduate radiography problem-based learning group. Journal of Medical Imaging and Radiation Sciences. 2015;46(3):280-6. doi: 10.1016/j.jmir.2015.06.007.
139. Ramirez-Velez R, Bagur-Calafat MC, Correa-Bautista JE, Girabent-Farres M. Barriers against incorporating evidence-based practice in physical therapy in Colombia: current state and factors associated. BMC Medical Education. 2015;15:220. PMID: 26655253.
140. Parasuraman S, Mueen Ahmed K, Hashim T, Muralidharan S, Kumar K, Ping W, et al. Knowledge about the availability of the pharmacist in the Nuclear Medicine Department: A questionnaire-based study among health-care professionals. Journal of Basic and Clinical Pharmacy. 2015;6(1):19-23. doi: 10.4103/0976-0105.145773.
141. Nason GJ, O'Kelly F, Kelly ME, Phelan N, Manecksha RP, Lawrentschuk N, et al. The emerging use of Twitter by urological journals. BJU International. 2015;115(3):486-90. PMID: 24925047.
142. McDonald JJ, Bisset C, Coleman MG, Speake D, Brady RR. Contemporary use of social media by consultant colorectal surgeons. Colorectal Disease. 2015;17(2):165-71. PMID: 25213268.
143. Kocemba P, Lasota M, Sroka NH, Feleszko W. Facebook-based medicine, or the doctor's professional image on the Internet. Pediatria i Medycyna Rodzinna. 2015;11(3):328-38. doi: 10.15557/PiMR.2015.0032.
144. Fox CS, Bonaca MA, Ryan JJ, Massaro JM, Barry K, Loscalzo J. A randomized trial of social media from Circulation. Circulation. 2015;131(1):28-33. PMID: 25406308.
145. Cabrini L, Esquinas A, Pasin L, Nardelli P, Frati E, Pintaudi M, et al. An International Survey on Noninvasive Ventilation Use for Acute Respiratory Failure in General Non-Monitored Wards. Respiratory Care. 2015;60(4):586-92. PMID: 103789612. Language: English. Entry Date: 20150427. Revision Date: 20150819. Publication Type: Journal Article. doi: 10.4187/respcare.03593.
146. McKay M, Sanko JS, Shekhter I, Birnbach DJ. Twitter as a tool to enhance student engagement during an interprofessional patient safety course. Journal of

Interprofessional Care. 2014;28(6):565-7. PMID: 103901380. Language: English. Entry Date: 20141015. Revision Date: 20160425. Publication Type: Journal Article. doi: 10.3109/13561820.2014.912618.
147. Croos S. The practice of clinical handover: a respite perspective. British Journal of Nursing. 2014;23(13):733-7. PMID: 103974458. Language: English. Entry Date: 20140715. Revision Date: 20150820. Publication Type: Journal Article. doi: 10.12968/bjon.2014.23.13.733.
148. Sönmez MO, Sevindik F. The effect of transformation in health on health personnel: To be family health personnel. TAF Preventive Medicine Bulletin. 2013;12(1):43-8. doi: 10.5455/pmb.1-1337859309.
149. Cain J, Scott DR, Tiemeier AM, Akers P, Metzger AH. Social media use by pharmacy faculty: Student friending, e-professionalism, and professional use. Currents in Pharmacy Teaching and Learning. 2013;5(1):2-8. doi: 10.1016/j.cptl.2012.09.002.
150. Lau AS. Hospital-based nurses' perceptions of the adoption of Web 2.0 tools for knowledge sharing, learning, social interaction and the production of collective intelligence. Journal of Medical Internet Research. 2011;13(4):e92. PMID: 22079851.
151. Henderson D, Carson-Stevens A, Bohnen J, Gutnik L, Hafiz S, Mills S. Check a Box. Save a Life: How student leadership is shaking up health care and driving a revolution in patient safety. Journal of patient safety. 2010;6(1):43-7. PMID: 22130303.
152. Yoshiura VT, Azevedo-Marques JM, Rzewuska M, Vinci ALT, Sasso AM, Miyoshi NSB, et al. A web-based information system for a regional public mental healthcare service network in Brazil. International Journal of Mental Health Systems. 2017;11(1). doi: 10.1186/s13033-016-0117-z.
153. Levine S, O'Mahony S, Baron A, Ansari A, Deamant C, Frader J, et al. Training the Workforce: Description of a Longitudinal Interdisciplinary Education and Mentoring Program in Palliative Care. Journal of Pain and Symptom Management. 2017;53(4):728-37. doi: 10.1016/j.jpainsymman.2016.11.009.
154. Thurtle N, Banks C, Cox M, Pain T, Furyk J. Free Open Access Medical Education resource knowledge and utilisation amongst Emergency Medicine trainees: A survey in four countries. African Journal of Emergency Medicine. 2016;6(1):12-7. doi: 10.1016/j.afjem.2015.10.005.
155. Praveen T, Karthick K, Thapasya M, Sai Preethika S. FlierMeet: An extension to online social networking site (OSNs). IIOAB Journal. 2016;7(9Special Issue):419-29.
156. Pitcock JJ, Barber KE, Theilman GD, Riche DM. Implementation of Twitter and Google Voice to simulate a pharmacy resident's day on-call for third-year pharmacy students. Currents in Pharmacy Teaching and Learning. 2016;8(6):804-10. doi:

10.1016/j.cptl.2016.08.022.
157. MacWalter G, McKay J, Bowie P. Utilisation of internet resources for continuing professional development: a cross-sectional survey of general practitioners in Scotland. BMC Medical Education. 2016;16:24. PMID: 26791566.
158. Kumar HR, Parthipan MRV. Smart portal application a complete app for student and staff management. International Journal of Pharmacy and Technology. 2016;8(4):21212-7.
159. du Plessis E. Caring presence in practice: facilitating an appreciative discourse in nursing. International Nursing Review. 2016;63(3):377-80. PMID: 27420641.
160. Ayadi MG, Bouslimi R, Akaichi J. A medical image retrieval scheme through a medical social network. Network Modeling and Analysis in Health Informatics and Bioinformatics. 2016;5(1). doi: 10.1007/s13721-016-0130-9.
161. Stinson J, Hussain A, Cafazzo JA, Gupta A, Hodgson D, Jamieson T, et al. A social networking tool for collaborative care for adolescents and young adults with cancer, caregivers, and health care professionals: A usability study. Pediatric Blood and Cancer. 2015;62:S192. doi: 10.1002/pbc.25715.
162. Lyngstad M, Hofoss D, Grimsmo A, Helleso R. Predictors for assessing electronic messaging between nurses and general practitioners as a useful tool for communication in home health care services: a cross-sectional study. Journal of Medical Internet Research. 2015;17(2):e47. PMID: 25691234.
163. Hogenbirk JC, French MG, Timony PE, Strasser RP, Hunt D, Pong RW. Outcomes of the northern Ontario school of medicine's distributed medical education programmes: Protocol for a longitudinal comparative multicohort study. BMJ Open. 2015;5(7). doi: 10.1136/bmjopen-2015-008246.
164. Chipps J, Pimmer C, Brysiewicz P, Walters F, Linxen S, Ndebele T, et al. Using mobile phones and social media to facilitate education and support for rural-based midwives in South Africa. Curationis. 2015;38(2):1500. PMID: 26842093.
165. Carter SC, Chiang A, Shah G, Kwan L, Montgomery JS, Karam A, et al. Video-based peer feedback through social networking for robotic surgery simulation: a multicenter randomized controlled trial. Annals of Surgery. 2015;261(5):870-5. PMID: 24887970.
166. Trinacty M, Farrell B, Schindel TJ, Sunstrum L, Dolovich L, Kennie N, et al. Learning and networking: Utilization of a primary care listserv by pharmacists. Canadian Journal of Hospital Pharmacy. 2014;67(5):343-52.
167. Kahlon M, Yuan L, Daigre J, Meeks E, Nelson K, Piontkowski C, et al. The use and significance of a research networking system. Journal of Medical Internet Research. 2014;16(2):e46. PMID: 24509520.
168. Heo JC, Han S. The effectiveness of streaming video with web-based text in

online course: Comparative study on three types of online instruction for Korean college students. Journal of Cyber Therapy and Rehabilitation. 2014;7(1):19.
169. Frisch N, Atherton P, Borycki E, Mickelson G, Cordeiro J, Novak Lauscher H, et al. Growing a professional network to over 3000 members in less than 4 years: evaluation of InspireNet, British Columbia's virtual nursing health services research network. J Med Internet Res. 2014 Feb 21;16(2):e49. PMID: 24566806. doi: 10.2196/jmir.3018.
170. Sharma D, Kumaresan K, Ashok B. Internet use among medical students in an institution in South India. Indian Journal of Public Health Research and Development. 2013;4(2):110-4. doi: 10.5958/j.0976-5506.4.2.024.
171. Rothman BS, Dexter F, Epstein RH. Communication latencies of Apple push notification messages relevant for delivery of time-critical information to anesthesia providers. Anesthesia & Analgesia. 2013;117(2):398-404. PMID: 23757478.
172. Takao H, Murayama Y, Ishibashi T, Karagiozov KL, Abe T. A new support system using a mobile device (smartphone) for diagnostic image display and treatment of stroke. Stroke. 2012;43(1):236-9. PMID: 21998052.
173. Nordqvist C, Hanberger L, Timpka T, Nordfeldt S. Health professionals' attitudes towards using a Web 2.0 portal for child and adolescent diabetes care: qualitative study. J Med Internet Res. 2009 Apr 06;11(2):e12. PMID: 19403464. doi: 10.2196/jmir.1152.
174. Ramkumar PN, La T, Fisch E, Fabricant PD, White AE, Jones KJ, et al. Integrating Social Media and Anterior Cruciate Ligament Surgery: An Analysis of Patient, Surgeon, and Hospital Use. Arthroscopy - Journal of Arthroscopic and Related Surgery. 2017;33(3):579-85. doi: 10.1016/j.arthro.2016.08.021.
175. Varshney AM, Shukla AK, Ahmad S, Mattas S. An interventional study on smartphones usage pattern among dental student of district Meerut, (UP). Indian Journal of Public Health Research and Development. 2017;8(1):68-72. doi: 10.5958/0976-5506.2017.00015.8.
176. Willemse JJ. Undergraduate nurses reflections on Whatsapp use in improving primary health care education. Curationis. 2015;38(2):1512. PMID: 26304053.
177. Chang JB, Woo SL, Cederna PS. Worth the "Likes"? The Use of Facebook among Plastic Surgeons and Its Perceived Impact. Plastic & Reconstructive Surgery. 2015;135(5):909e-18e. PMID: 25919273.
