# Supplementary material for: Use of Social Network Sites for Communication Among Health Professionals: Systematic Review
Source: J Med Internet Res. 2018 Mar 28;20(3):e117. doi: 10.2196/jmir.8382 (PMC5895921; doi:10.2196/jmir.8382)
Supplement: Multimedia Appendix 3 [file jmir_v20i3e117_app3.pdf]

Multimedia Appendix 3: Quality of the reviewed studies

<u>Quality of qualitative and mixed-methods studies (n=20)</u>

Critical Appraisal Skills Programme (CASP) Qualitative Research Checklist 13.03.17[1]
(1) Was there a clear statement of the aims of the research?
(2) Is a qualitative methodology appropriate?
(3) Was the research design appropriate to address the aims of the research?
(4) Was the recruitment strategy appropriate to the aims of the research?
(5) Was the data collected in a way that addressed the research issue?
(6) Has the relationship between researcher and participants been adequately considered?
(7) Have ethical issues been taken into consideration?
(8) Was the data analysis sufficiently rigorous?
(9) Is there a clear statement of findings?
(10) How valuable is the research?

**First Author**	**(1)**	**(2)**	**(3)**	**(4)**	**(5)**	**(6)**	**(7)**	**(8)**	**(9)**	**(10)**	**Rating**
Barry et al 2015[2]	yes	yes	yes	yes	yes	can't tell	yes	yes	yes	yes	Satisfactory
Benetoli et al 2016[3]	yes	yes	yes	can't tell	yes	can't tell	yes	yes	yes	yes	Satisfactory
Dong et al 2015[4]	yes	yes	yes	yes	yes	can't tell	yes	can't tell	yes	yes	Satisfactory
Dieleman et al 2013[5]	yes	yes	yes	yes	yes	yes	yes	yes	yes	yes	Satisfactory
Flynn et al 2017[6]	yes	yes	yes	yes	yes	yes	yes	yes	yes	yes	Satisfactory
Goff et al 2016[7]	yes	yes	yes	yes	yes	yes	yes	can't tell	yes	yes	Satisfactory
Gruzd et al 2013[8]	yes	yes	yes	yes	yes	yes	can't tell	yes	yes	yes	Satisfactory
Gulacti et al 2016[9]	yes	yes	yes	yes	yes	yes	yes	can't tell	yes	yes	Satisfactory
Johnston et al 2015[10]	yes	yes	yes	yes	yes	yes	yes	yes	yes	yes	Satisfactory
Lipp et al 2014[11]	yes	yes	yes	yes	can't tell	can't tell	yes	yes	yes	yes	Satisfactory
Loeb 2014[12]	yes	yes	yes	yes	yes	yes	yes	yes	yes	yes	Satisfactory
Lofters et al 2016[13]	yes	yes	yes	yes	yes	yes	yes	yes	yes	yes	Satisfactory
Maisonneuve et al 2015[14]	yes	can't tell	yes	yes	can't tell	yes	yes	yes	yes	yes	Satisfactory
Mawdsley et al 2015[15]	yes	yes	yes	yes	yes	can't tell	yes	yes	yes	yes	Satisfactory
Morley 2014[16]	yes	yes	yes	yes	yes	yes	yes	can't tell	yes	yes	Satisfactory

**First Author**	**(1)**	**(2)**	**(3)**	**(4)**	**(5)**	**(6)**	**(7)**	**(8)**	**(9)**	**(10)**	**Rating**
Raiman et al 2017[17]	yes	yes	yes	yes	yes	can’t tell	yes	yes	yes	yes	Satisfactory
Reames et al 2016[18]	yes	yes	yes	yes	yes	can’t tell	yes	yes	yes	yes	Satisfactory
Wang et al 2013[19]	yes	yes	yes	yes	yes	can’t tell	yes	can’t tell	yes	yes	Satisfactory
Wani et al 2013[20]	yes	can’t tell	yes	yes	can’t tell	can’t tell	yes	can’t tell	yes	yes	Fair
Winandy et al 2016[21]	yes	yes	yes	yes	yes	yes	yes	yes	yes	yes	Satisfactory

Quality of quantitative studies (n=13)

The Quality Assessment Tool for Observational Cohort and Cross-Sectional Studies [22]

1. Was the research question or objective in this paper clearly stated?
2. Was the study population clearly specified and defined?
3. Was the participation rate of eligible persons at least 50%?
4. Were all the subjects selected or recruited from the same or similar populations (including the same time period)? Were inclusion and exclusion criteria for being in the study pre-specified and applied uniformly to all participants?
5. Was a sample size justification, power description, or variance and effect estimates provided?
6. For the analyses in this paper, were the exposure(s) of interest measured prior to the outcome(s) being measured?
7. Was the timeframe sufficient so that one could reasonably expect to see an association between exposure and outcome if it existed?
8. For exposures that can vary in amount or level, did the study examine different levels of the exposure as related to the outcome (e.g., categories of exposure, or exposure measured as continuous variable)?
9. Were the exposure measures (independent variables) clearly defined, valid, reliable, and implemented consistently across all study participants?
10. Was the exposure(s) assessed more than once over time?
11. Were the outcome measures (dependent variables) clearly defined, valid, reliable, and implemented consistently across all study participants?
12. Were the outcome assessors blinded to the exposure status of participants?
13. Was loss to follow-up after baseline 20% or less?
14. Were key potential confounding variables measured and adjusted statistically for their impact on the relationship between exposure(s) and outcome(s)?

First Author	1.	2.	3.	4.	5.	6.	7.	8.	9.	10.	11.	12.	13.	14.	Rating
Cain et al 2013[23]	yes	yes	no	yes	NA	yes	yes	yes	yes	yes	yes	yes	NA	yes	Good
Deen et al 2013[24]	yes	yes	no	yes	NA	yes	yes	yes	yes	yes	yes	yes	NA	yes	Good
Desselle 2017[25]	yes	yes	yes	yes	NA	yes	yes	yes	yes	yes	yes	yes	NA	yes	Good
Fuoco et al 2015[26]	yes	yes	no	yes	NA	yes	yes	yes	yes	yes	yes	yes	NA	yes	Good
Ganasegeran et al 2017[27]	yes	yes	yes	yes	NA	yes	yes	yes	yes	yes	yes	yes	NA	yes	Good
Keller et al 2014[28]	yes	yes	no	yes	NA	yes	yes	yes	yes	yes	yes	yes	NA	yes	Good
Kostka-Rokosz 2014[29]	yes	yes	yes	yes	yes	yes	yes	yes	yes	yes	yes	yes	Yes	yes	Good
Narayanaswami et al 2015[30]	yes	yes	no	yes	NA	yes	yes	yes	yes	yes	yes	yes	NA	yes	Good
Nikiphorou et al 2016[31]	yes	yes	NR	yes	NA	yes	yes	yes	yes	yes	yes	yes	NA	yes	Good
Patel et al 2017[32]	yes	yes	NR	yes	NA	yes	yes	yes	yes	yes	yes	yes	NA	yes	Good
Siegal et al 2016[33]	yes	yes	no	yes	NA	yes	yes	yes	yes	yes	yes	yes	NA	yes	Good
Stevens et al 2012[34]	yes	yes	no	yes	NA	yes	yes	yes	yes	yes	yes	yes	NA	yes	Good
Wang et al 2012[35]	yes	yes	no	yes	NA	yes	yes	yes	yes	yes	yes	yes	NA	yes	Good

Key: NA, not applicable; NR, not reported

References

1. Critical Appraisal Skills Programme. 2017. CASP Tools & Checklists. http://www.casp-uk.net/casp-tools-checklists. Archived at: http://www.webcitation.org/6rYs9TujA
2. Barry AR, Pearson GJ. Professional use of social media by pharmacists. Canadian Journal of Hospital Pharmacy. 2015;68(1):22-7. PMID: 25762816
3. Benetoli A, Chen TF, Schaefer M, Chaar BB, Aslani P. Professional Use of Social Media by Pharmacists: A Qualitative Study. Journal of Medical Internet Research. 2016;18(9):e258. PMID: 27663570.
4. Dong C, Cheema M, Samarasekera D, Rajaratnam V. Using LinkedIn for Continuing Community of Practice Among Hand Surgeons Worldwide. Journal of Continuing Education in the Health Professions. 2015;35(3):185-91. PMID: 26378424.
5. Dieleman C, Duncan EA. Investigating the purpose of an online discussion group for health professionals: a case example from forensic occupational therapy. BMC Health Services Research. 2013;13:253. PMID: 23822895.
6. Flynn S, Hebert P, Korenstein D, Ryan M, Jordan WB, Keyhani S. Leveraging social media to promote evidence-based continuing medical education. PLoS ONE. 2017;12(1). doi: 10.1371/journal.pone.0168962.
7. Goff DA, Jones C, Toney B, Nwomeh BC, Bauer K, Ellison EC. Use of Twitter to Educate and Engage Surgeons in Infectious Diseases and Antimicrobial Stewardship. Infectious Diseases in Clinical Practice. 2016;24(6):324-7. doi: 10.1097/IPC.0000000000000440.
8. Gruzd A, Haythornthwaite C. Enabling community through social media. Journal of Medical Internet Research. 2013;15(10):e248. PMID: 24176835.
9. Gulacti U, Lok U, Hatipoglu S, Polat H. An Analysis of WhatsApp Usage for Communication Between Consulting and Emergency Physicians. Journal of Medical Systems. 2016;40(6). doi: 10.1007/s10916-016-0483-8.
10. Johnston MJ, King D, Arora S, Behar N, Athanasiou T, Sevdalis N, et al. Smartphones let surgeons know WhatsApp: an analysis of communication in emergency surgical teams. Am J Surg. 2015 Jan;209(1):45-51. PMID: 25454952. doi: 10.1016/j.amjsurg.2014.08.030.
11. Lipp A, Davis RE, Peter R, Davies JS. The use of social media among health care professionals within an online postgraduate diabetes diploma course. Practical Diabetes. 2014;31(1):14-7a. doi: 10.1002/pdi.1821.
12. Loeb S, Bayne CE, Frey C, Davies BJ, Averch TD, Woo HH, et al. Use of social media in urology: data from the American Urological Association (AUA). BJU International. 2014;113(6):993-8. PMID: 24274744.
13. Lofters AK, Slater MB, Angl EN, Leung FH. Facebook as a tool for communication, collaboration, and informal knowledge exchange among members of a multisite family health team. Journal of Multidisciplinary Healthcare. 2016;9:29-34. doi: 10.2147/JMDH.S94676.
14. Maisonneuve H, Chambe J, Lorenzo M, Pelaccia T. How do general practice residents use social networking sites in asynchronous distance learning? BMC Medical Education. 2015;15:154.

PMID: 26391989.
15. Mawdsley A, Schafheutle EI. Using Facebook to support learning and exam preparation in a final-year undergraduate pharmacy clinical therapeutics module. Currents in Pharmacy Teaching and Learning. 2015;7(6):869-75. doi: 10.1016/j.cptl.2015.08.010.
16. Morley DA. Supporting student nurses in practice with additional online communication tools. Nurse Education in Practice. 2014;14(1):69-75. PMID: 104006548. Language: English. Entry Date: 20140128. Revision Date: 20150820. Publication Type: Journal Article. doi: 10.1016/j.nepr.2013.06.005.
17. Raiman L, Antbring R, Mahmood A. WhatsApp messenger as a tool to supplement medical education for medical students on clinical attachment. BMC Medical Education. 2017;17(1):7. PMID: 28061777.
18. Reames BN, Sheetz KH, Englesbe MJ, Waits SA. Evaluating the Use of Twitter to Enhance the Educational Experience of a Medical School Surgery Clerkship. Journal of Surgical Education. 2016;73(1):73-8. doi: 10.1016/j.jsurg.2015.08.005.
19. Wang T, Wang F, Shi L. The use of microblog-based case studies in a pharmacotherapy introduction class in China. BMC Medical Education. 2013;13:120. PMID: 24010945.
20. Wani SA, Rabah SM, Alfadil S, Dewanjee N, Najmi Y. Efficacy of communication amongst staff members at plastic and reconstructive surgery section using smartphone and mobile WhatsApp. Indian Journal of Plastic Surgery. 2013;46(3):502-5. PMID: 24459338.
21. Winandy M, Kostkova P, de Quincey E, St Louis C, Szomszor M. Follow #eHealth2011: Measuring the Role and Effectiveness of Online and Social Media in Increasing the Outreach of a Scientific Conference. Journal of Medical Internet Research. 2016;18(7):e191. PMID: 27436012.
22. National Heart, Lung, and Blood Institute. 2014. Quality Assessment Tool for Observational Cohort and Cross-Sectional Studies - NHLBI, NIH. https://www.nhlbi.nih.gov/health-pro/guidelines/in-develop/cardiovascular-risk-reduction/tools/cohort . Archived at: http://www.webcitation.org/6rYsddMPI
23. Cain J, Scott DR, Tiemeier AM, Akers P, Metzger AH. Social media use by pharmacy faculty: Student friending, e-professionalism, and professional use. Currents in Pharmacy Teaching and Learning. 2013;5(1):2-8. doi: 10.1016/j.cptl.2012.09.002.
24. Deen SR, Withers A, Hellerstein DJ. Mental health practitioners' use and attitudes regarding the Internet and social media. Journal of Psychiatric Practice. 2013;19(6):454-63. PMID: 24241499.
25. Desselle SP. The use of Twitter to facilitate engagement and reflection in a constructionist learning environment. Currents in Pharmacy Teaching and Learning. 2017;9(2):185-94. doi: 10.1016/j.cptl.2016.11.016.
26. Fuoco M, Leveridge MJ. Early adopters or laggards? Attitudes toward and use of social media among urologists. BJU International. 2015;115(3):491-7. PMID: 24981237.
27. Ganasegeran K, Renganathan P, Rashid A, Al-Dubai SAR. The m-Health revolution: Exploring perceived benefits of WhatsApp use in clinical practice. International Journal of Medical Informatics.

2017;97:145-51. doi: 10.1016/j.ijmedinf.2016.10.013.
28. Keller B, Labrique A, Jain KM, Pekosz A, Levine O. Mind the gap: social media engagement by public health researchers. Journal of Medical Internet Research. 2014;16(1):e8. PMID: 24425670.
29. Kostka-Rokosz MD, Camiel LD, McCloskey WW. Pharmacy students' perception of the impact of a Facebook-delivered health news service – a two-year analysis. Currents in Pharmacy Teaching and Learning. 2014;6(4):471-7. doi: 10.1016/j.cptl.2014.04.007.
30. Narayanaswami P, Gronseth G, Dubinsky R, Penfold-Murray R, Cox J, Bever C, Jr., et al. The Impact of Social Media on Dissemination and Implementation of Clinical Practice Guidelines: A Longitudinal Observational Study. Journal of Medical Internet Research. 2015;17(8):e193. PMID: 26272267.
31. Nikiphorou E, Studenic P, Ammitzbøll CG, Canavan M, Jani M, Ospelt C, et al. Social media use among young rheumatologists and basic scientists: Results of an international survey by the Emerging EULAR Network (EMEUNET). Annals of the Rheumatic Diseases. 2016. doi: 10.1136/annrheumdis-2016-209718.
32. Patel SS, Hawkins CM, Rawson JV, Hoang JK. Professional Social Networking in Radiology: Who Is There and What Are They Doing? Academic Radiology. 2017. doi: 10.1016/j.acra.2016.09.026.
33. Siegal G, Dagan E, Wolf M, Duvdevani S, Alon EE. Medical Information Exchange: Pattern of Global Mobile Messenger Usage among Otolaryngologists. Otolaryngology - Head and Neck Surgery (United States). 2016;155(5):753-7. doi: 10.1177/0194599816656178.
34. Stevens RJG, Hamilton NM, O'Donoghue JM, Davies MP. The use of the Internet and social software by plastic surgeons. European Journal of Plastic Surgery. 2012;35(10):747-55. doi: 10.1007/s00238-011-0681-z.
35. Wang AT, Sandhu NP, Wittich CM, Mandrekar JN, Beckman TJ. Using social media to improve continuing medical education: a survey of course participants. Mayo Clin Proc. 2012 Dec;87(12):1162-70. PMID: 23141117. doi: 10.1016/j.mayocp.2012.07.024.
